# SLC20A2-related primary familial brain calcification with purely acute psychiatric symptoms: a case report

**DOI:** 10.1186/s12883-022-02798-9

**Published:** 2022-07-18

**Authors:** Weiting Bu, Lijing Hou, Meijia Zhu, Renyun Zhang, Xiaoyu Zhang, Xiao Zhang, Jiyou Tang, Xiaomin Liu

**Affiliations:** 1 Department of Neurology, Shandong Provincial Qianfoshan Hospital, Weifang Medical University, 250014 Jinan, People’s Republic of China; 2 Institute of Traditional Chinese Medicine Pharmacology, Shandong Academy of Chinese Medicine, 250014 Jinan, People’s Republic of China; 3 Department of Neurology, the First Affiliated Hospital of Shandong First Medical University & Shandong Provincial Qianfoshan Hospital, 16766 Jingshi Road, 250014 Jinan, Shandong People’s Republic of China; 4 Department of Psychiatry, Shandong Mental Health Center Affiliated to Shandong University, 250014 Jinan, People’s Republic of China

**Keywords:** Primary familial brain calcification, Psychiatric symptoms, *SLC20A2* gene, Mutation, Case report

## Abstract

**Background:**

Primary familial brain calcification (PFBC) is a rare inherited neurological disorder characterized by bilateral basal ganglia calcification with a series of motor and nonmotor symptoms. Mutations in the *SLC20A2* gene, encoding the PiT2 protein, are the major cause of the disease. Here, we report a Chinese PFBC family carrying a *SLC20A2* gene mutation, and the proband presented with purely acute psychiatric symptoms, which has been rarely reported in this disease.

**Case presentation:**

A 38-year-old woman was hospitalized due to disorganized speech; disordered thought contents; disorganized behaviour; emotional instability and lability; and grandiose words, actions and facial expressions. Brain computerized tomography (CT) revealed calcification in the basal ganglia; cerebellar dentate nuclei; and subcortical, periventricular, and deep white matter regions in she and her family members. Through mutation analysis, a heterozygous truncating mutation, c.1723G > T, p.(Glu575*), was identified in the *SLC20A2* gene in this family. Thus, this patient was diagnosed with genetically confirmed PFBC, and she responded well to a low dose of antipsychotic drugs. The penetrance of the disease in this family was only 33%, which was significantly lower than that in most families carrying *SLC20A2* gene mutations.

**Conclusions:**

Patients with *SLC20A2-*related PFBC might present with psychiatric symptoms alone, and the penetrance of the disease may be quite low, which adds to the clinical heterogeneity of the disease.

**Supplementary Information:**

The online version contains supplementary material available at 10.1186/s12883-022-02798-9.

## Background

**Fig. 1:**
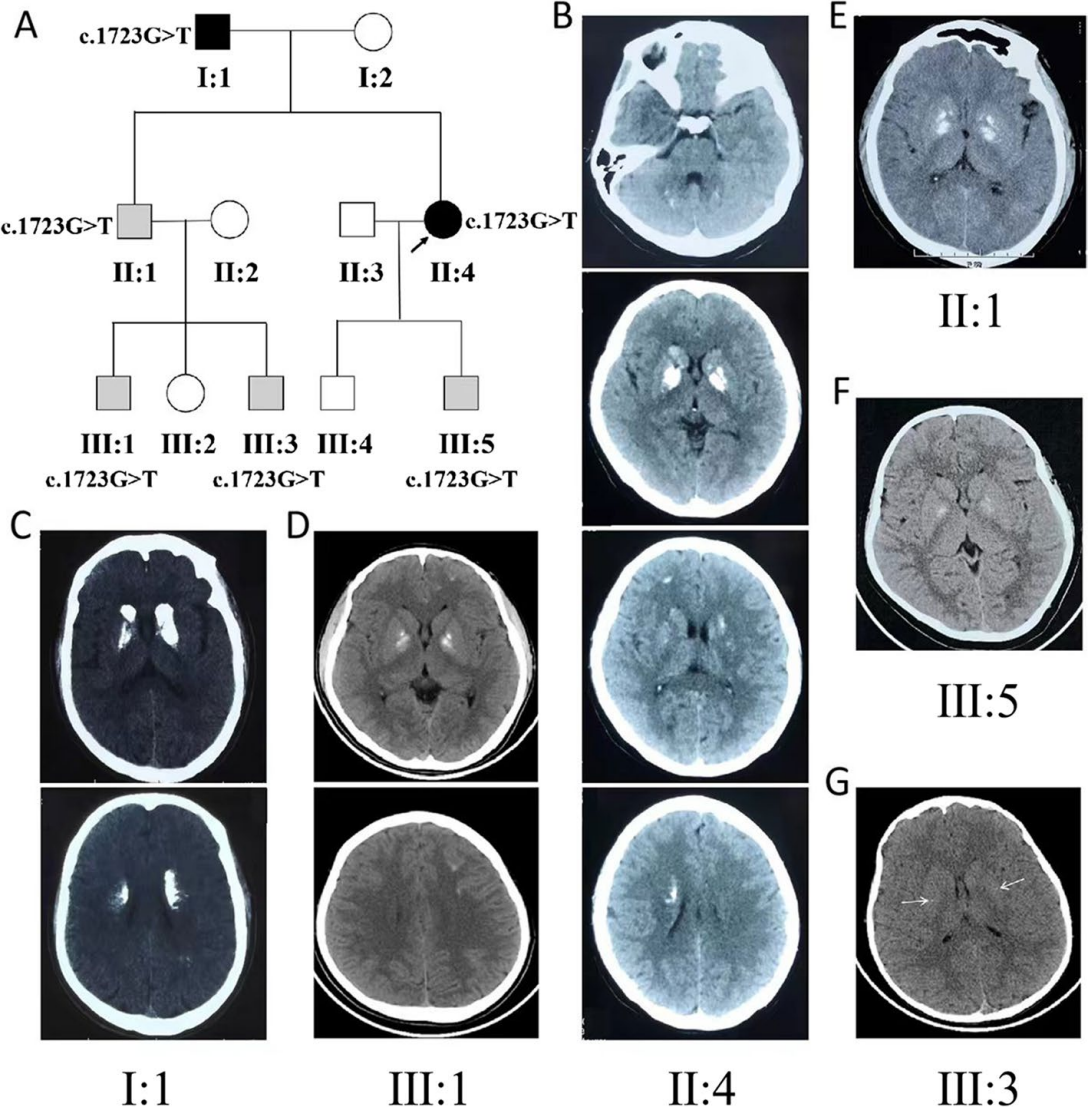
Pedigree diagram and brain computed tomography (CT). **A** Pedigree of the family with primary familial brain calcification (PFBC). The proband is indicated by an arrow. The black shaded symbols indicate the symptomatic patients with PFBC. The grey shaded symbols indicate the asymptomatic patients with PFBC. **B** Patient II: 4, brain CT showed calcification in the cerebellum, basal ganglia, subcortical and deep white matter. **C** Patient I: 1, brain CT showed calcification in the basal ganglia, white matter adjacent to the lateral ventricle and subcortical white matter. **D** Patient III: 1, brain CT showed calcification in the basal ganglia and subcortical white matter. **E-F** Patient II: 1 and patient III: 5, brain CT showed symmetrical calcification only in the bilateral basal ganglia. **G** Patient III: 3, brain CT showed mild unsymmetrical scattered punctate calcifications only in the basal ganglia (arrows)

Primary familial brain calcification (PFBC) is a clinically and genetically heterogeneous neurodegenerative disorder with characteristic bilateral calcification of the basal ganglia. To date, at least six causative genes have been identified, including the *SLC20A2* gene, *PDGFRB* gene, *PDGFB* gene, *XPR1* gene, *MYORG* gene, and *JAM2* gene, among which mutations in the *SLC20A2* gene account for approximately 61% of PFBC cases [1]. Here, we describe a Chinese family with *SLC20A2-*related autosomal-dominant PFBC (Fig. 1 A). The proband presented with acute psychiatric symptoms as the sole manifestation, and these symptoms were treated effectively with a low dose of quetiapine.

## Case presentation

A 38-year-old Chinese woman (patient II: 4)was hospitalized due to acute psychiatric manifestations that started four days prior after she had a quarrel with her colleague at work. She presented with disorganized speech, disorganized behaviour, and emotional instability and lability. Initially, she roamed around the whole day and became overtalkative but without a clear theme. She spoke in a heightened voice and displayed a euphoric mood and disordered dance. When others interrupted her speaking, she became irritable. Gradually, she developed abnormal thoughts and said, “There is a devil threatening to hurt me”. She also developed insomnia and got up late at night to read the Bible and kneel down to pray for God’s favour and protection. She thought that none of her family members cared about her, and she subsequently often cried loudly. There was no consciousness disorder, anxiety, depression, hallucination, impulsive or aggressive behaviour, suicide or self-injurious behaviour. There were also no headaches, dizziness, epilepsy, cognitive impairments, extrapyramidal symptoms, or cerebellar ataxic symptoms. No psychiatric illness, intellectual disability, or organic medical disorders were reported in her personal and family history except for her older son, who suffered from hypoxic-ischaemic encephalopathy at birth and presented with psychomotor retardation. The findings of a neurological examination were normal. Psychiatric examination revealed increased psychomotor activity; increased speech production with accelerated tempo; disordered thought contents; disorganized communication; and grandiose words, actions and facial expressions. An instability and lability of the mood, feelings and emotions; a difficulty in sustaining attention; decreased wilful actions; a lack of cognitive and critical-thinking ability about her condition; and a disturbance of self-consciousness were observed. Her emotional response was inharmonious with her inner experience and the external surroundings.

Her serum calcium, phosphate, vitamin D, and parathyroid hormone levels were normal. Her 24-hour video-electroencephalography results were also normal. Brain computerized tomography (CT) revealed bilateral symmetrical calcification in the basal ganglia and cerebellar dentate nuclei. The subcortical and periventricular white matter were also affected (Fig. 1B). Mild to severe similar calcification was also detected in the brain of her father, aged 67 years (patient I: 1) (Fig. 1 C); her older brother’s older son, aged 13 years (patient III: 1) (Fig. 1D); her older brother, aged 41 years (patient II: 1) (Fig. 1E); her younger son, aged 12 years (patient III: 5) (Fig. 1 F); and her older brother’s younger son, aged five years (patient III: 3) (Fig. 1G). All of these family members were asymptomatic, and the findings of neurological examinations were normal, except for her father, who complained of intermittent headaches over the last 10 years. Her mother, aged 65 years, and her older brother’s daughter, aged seven years, had no calcification in the brain. A brain CT of her older son, aged 16 years, showed abnormal changes related to hypoxic-ischaemic encephalopathy at birth. An additional file shows these findings in more detail [see Additional file 1].

**Fig. 2:**
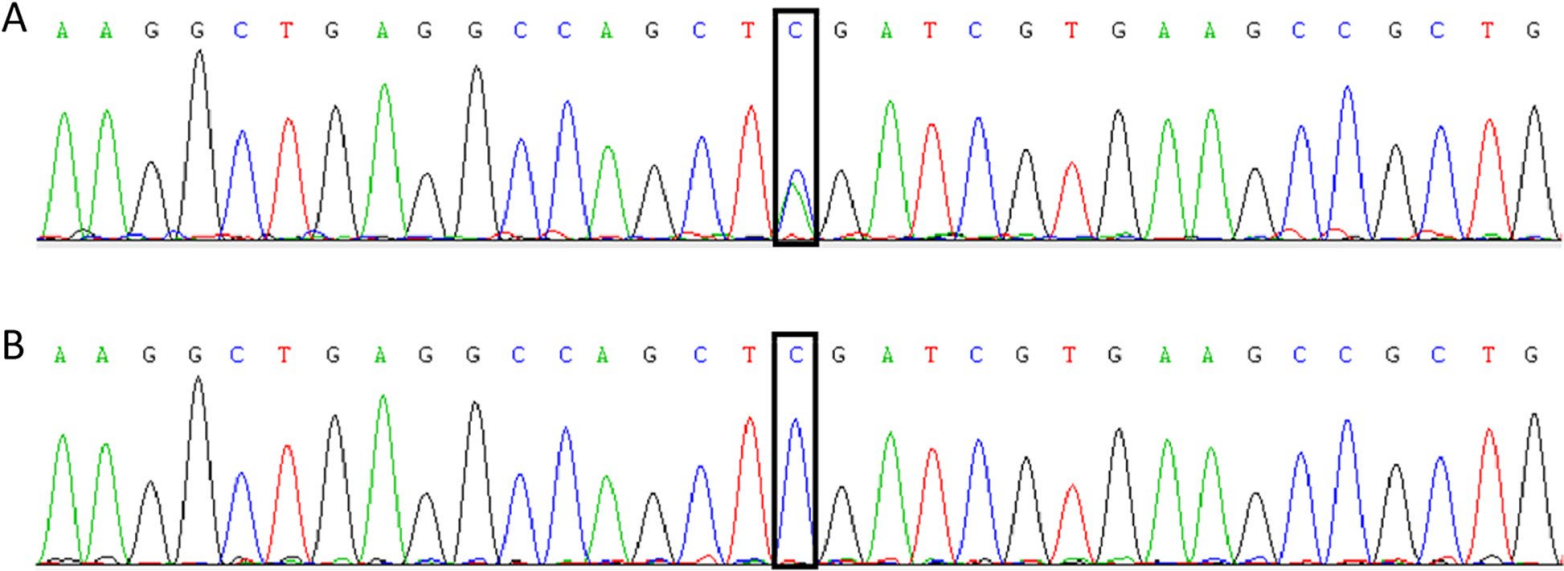
DNA sequencing results. **A** The heterozygous *SLC20A2* gene mutation, c.1723G > T, in this family. Black frames indicate the c.1723 nucleotide. **B** Wild-type SLC20A2 gene sequence

Informed consent was obtained, and the total DNA of peripheral blood samples extracted from nine participants was analysed. Whole-exome sequencing was performed on the proband to assess the whole exome, and Sanger sequencing was used to confirm the specific *SLC20A2* gene mutation in all participants. A heterozygous mutation, c.1723G > T, p.(Glu575*), was discovered in exon 10 of the *SLC20A2* gene in this family (Fig. 2 A, B). This mutation was found in two symptomatic patients (patients I: 1 and II: 4) and four asymptomatic patients (patients II: 1, III: 1, III: 3 and III: 5 ) and was absent in family members I: 2, III: 2 and III: 4. An additional file shows these findings in more detail [see Additional file 2]. The mutation segregated perfectly with the imaging findings. This mutation had already been submitted to ClinVar and classified as pathogenic (information available on ClinVar [https://www.ncbi.nlm.nih.gov/clinvar/RCV00062025/]). Therefore, these individuals were diagnosed as having genetically confirmed PFBC.

The proband was treated with a low dose of quetiapine (200 mg/day). Her psychiatric symptoms completely resolved in ten days, and she was discharged from the hospital. After one month of maintenance treatment on the same dose, the patient terminated the medication at her own discretion. She responded well to a low-dose quetiapine without any side effects. At the one-year follow-up, no recurrence of the psychiatric symptoms was reported.

## Discussion and conclusions

Parkinsonism, speech disturbance, cognitive deficits, headache, and depression are the major manifestations of PFBC [1]. However, the proband in our study displayed purely psychiatric symptoms, which have been rarely reported in PFBC, especially in genetically confirmed cases. In 2013, Nicolas et al. [2] described the case of a 39-year-old PFBC patient who demonstrated purely auditory hallucinations and delusions, which were responsive to antipsychotics. In 2018, another patient with PFBC was reported to have manic symptoms alone [3]. However, the PFBC of these two patients were not genetically confirmed. Recently, Uno et al. [4] reported the case of a PFBC patient with a *SLC20A2* gene mutation who presented with purely recurrent psychiatric symptoms in a 10-year follow-up. These cases, including ours, raise the question that a putative purely psychiatric presentation of the disease would be a novel clinical finding. However, in 2021, Balck et al. [1] reviewed the clinical features of 349 patients with genetically confirmed symptomatic PFBC, including 191 patients with *SLC20A2*- linked PFBC. Psychosis was observed in only 9% and 9.4% of these patients, respectively. Furthermore, the psychiatric symptoms presented by these patients are commonly associated with neurological presentations in most cases [1, 4]. In addition, in 2016, the case of a white female carrying the same mutation identified in our study was reported. The patient presented with ataxia, anxiety, jaw pain, obsessive-compulsive disorder, brisk reflexes, coordination abnormalities, and basal ganglia calcifications, rather than psychiatric manifestation alone (information available on ClinVar [https://www.ncbi.nlm.nih.gov/clinvar/variation/522850/?new_evidence=true]). Psychotic disorders are frequent in the general population, and the median point, 12-month and lifetime prevalence rates for persons are 3.89, 4.03 and 7.49 per 1000, respectively [5]. Psychotic symptoms or psychotic-like experiences are more frequent, and the median prevalence rate and the median 1-year incidence rate for persons in the general population are approximately 5% and 3%, respectively [6]. Moreover, carriers of SLC20A2 mutations related to PFBC could be asymptomatic owing to the low penetrance of the disease of approximately only 60% [1]. For example, the other family members carrying the mutation in the current family showed no psychosis, and at the one-year follow-up after terminating the antipsychotic drug, the proband did not report a recurrence of the aforementioned psychiatric symptoms. Thus, the association of purely psychiatric symptoms and PFBC in our study may be merely coincidental. Longitudinal follow-up of the current family is required to determine whether the psychiatric symptoms relapse in the proband since stopping the medication and whether other neuropsychiatric and motor symptoms develop in the course of the disease in the family members, especially in the asymptomatic carriers. In clinical practice, causative gene mutation analysis should be performed in patients with pure psychosis and bilateral symmetrical basal ganglia calcification to genetically confirm diagnoses of PFBC.

PFBC may be inherited in an autosomal-dominant or autosomal-recessive pattern. However, due to the reduced penetrance, the inheritance pattern is often difficult to confirm only by taking the family history. Many asymptomatic individuals are diagnosed with PFBC by brain CT scan or gene mutation analysis. The current family showed an autosomal-dominant inheritance pattern. Including the proband, six individuals showed characteristic brain calcifications. However, four of these individuals were asymptomatic. The penetrance (33%) was significantly lower than that in most *SLC20A2* gene mutation carriers (approximately 60%) [1]. This low penetrance may be due to three of the individuals being younger than the age of onset of clinical manifestations at the time of neuroimaging (aged 5, 12, and 13 years, respectively), the median of which was reported to be 47 years in clinically affected *SLC20A2* gene mutation carriers [1]. These three individuals also showed relatively mild calcifications in the brain, especially patient III: 3, who was only aged 5 years.

By sequencing, a reported *SLC20A2* gene truncating mutation, c.1723G > T, p.(Glu575*), was identified in the current family. This mutation is predicted to cause the substitution of Glu575 with a new stop codon in the exon 10 of *SLC20A2* gene and is predicted to encode a truncated and dysfunctional protein reduced by 78 amino acids compared with the wild-type protein. Recently, Balck et al. [1] reviewed the cases of 167 index patients carrying *SLC20A2* gene mutations reported in previous studies and found that missense mutations were the most common mutation type (47%), followed by frameshift (18%), nonsense (16%), and splice site variations (10%). The c.1723G > A, p.( Glu575Lys) mutation was the most frequent mutation and was found five times. This mutation was at the same gene locus as the mutation found in the current family, suggesting that this site is a mutation hotspot in the *SLC20A2* gene. The *SLC20A2* gene encodes the 652 amino acid long type-III sodium-dependent phosphate transporter 2 (PiT2), which is a membrane protein with 12 transmembrane domains that plays a major role in the maintenance of cellular Pi homeostasis in the brain. Mutations in the *SLC20A2* gene caused Pi transport activity dysfunction of the PiT2 protein, subsequently leading to disrupted Pi homeostasis and ultimately inducing pathological calcium deposition in the brain and PFBC [7]. Previous studies have shown that both haploinsufficiency and dominant negative effects may be the likely mechanism responsible for *SLC20A2*-linked PFBC [7–9]. Thus, further studies should be performed to elucidate the mechanism of *SLC20A2* gene mutations causing PFBC.

The proband in our study was treated with a low dose of quetiapine and responded well without any side effects, which is consistent with other reported cases [2, 4, 10]. However, the prescription of antipsychotic drugs for PFBC patients should be done with caution and personalized, as these drugs may exacerbate extrapyramidal symptoms. The search for an agent to increase PiT2 protein activity is important for the future treatment of PFBC patients [7].

In conclusion, we describe a *SLC20A2*-related Chinese family with PFBC in which the proband presented with purely psychiatric symptoms. The penetrance of the disease in this family appeared to be quite low. This report provides novel insights into the clinical heterogeneity of PFBC. Further investigations are required to determine the genetic pathogenesis of PFBC and search for potential therapeutic targets of the disease.

## Supplementary Information

**Additional file 1.** Brain CT scan images. A-I Brain CT scan images of individuals I: 1, I: 2, II: 1, II: 4, III: 1, III: 2, III: 3, III: 4, and III: 5.

**Additional file 2.** DNA sequencing results. A-I DNA sequencing results of individuals I: 1, I: 2, II: 1, II: 4, III: 1, III: 2, III: 3, III: 4, and III: 5.

## Data Availability

All data have been presented within the manuscript and its supplementary information files.

## Abbreviations

PFBC: Primary familial brain calcification
CT: Computed tomography
PiT2: Type-III sodium-dependent phosphate transporter 2
