# Supplementary material for: SLC20A2-related primary familial brain calcification with purely acute psychiatric symptoms: a case report
Source: BMC Neurol. 2022 Jul 18;22:265. doi: 10.1186/s12883-022-02798-9 (PMC9290231; doi:10.1186/s12883-022-02798-9)

A. Brain CT scan images of individual I: 1

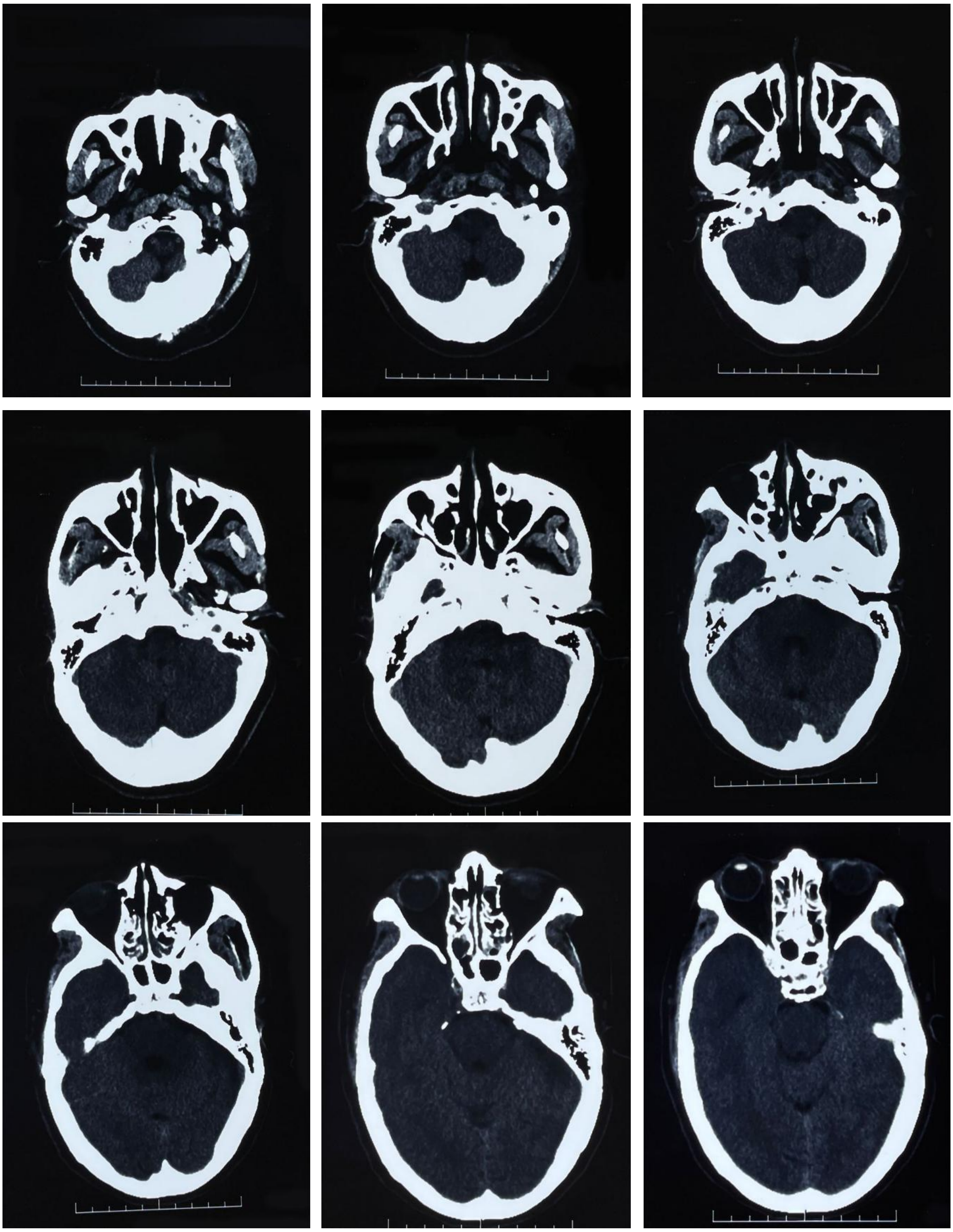

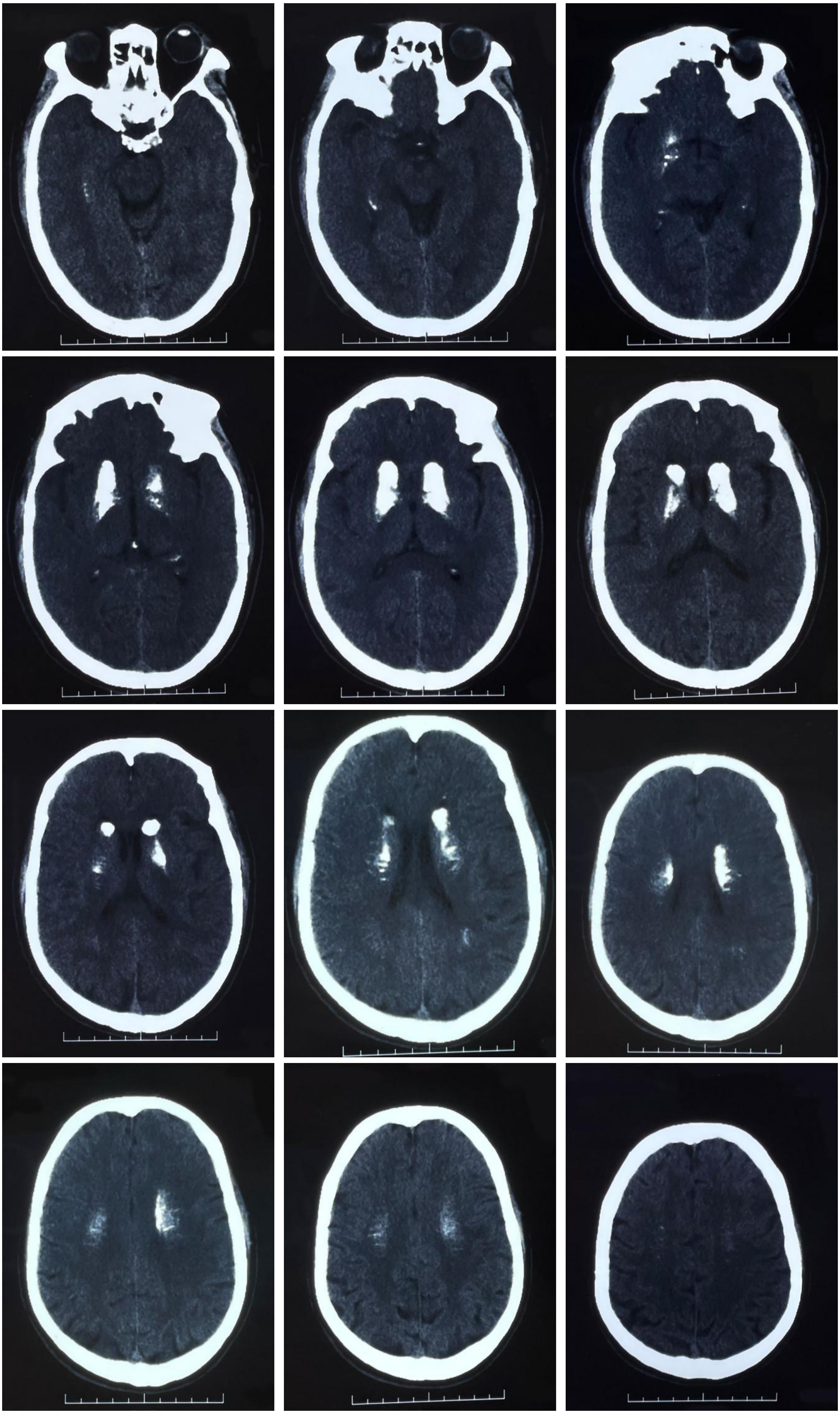

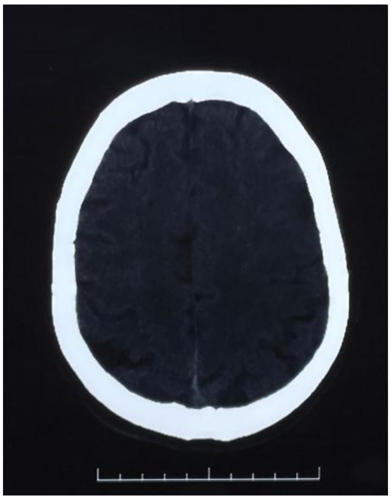
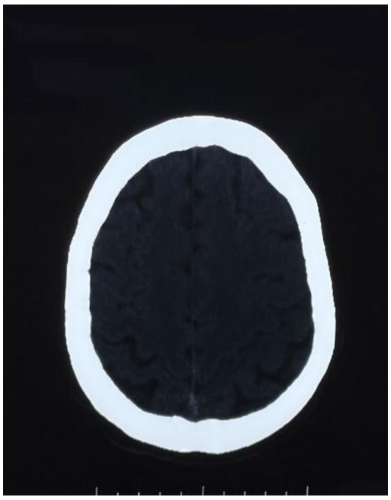
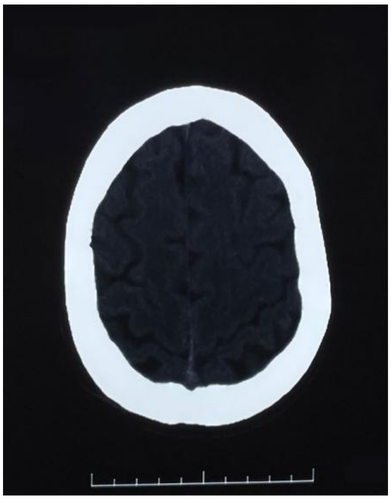
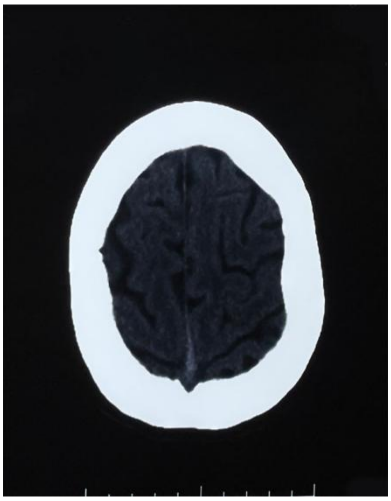

B. Brain CT scan images of individual I: 2

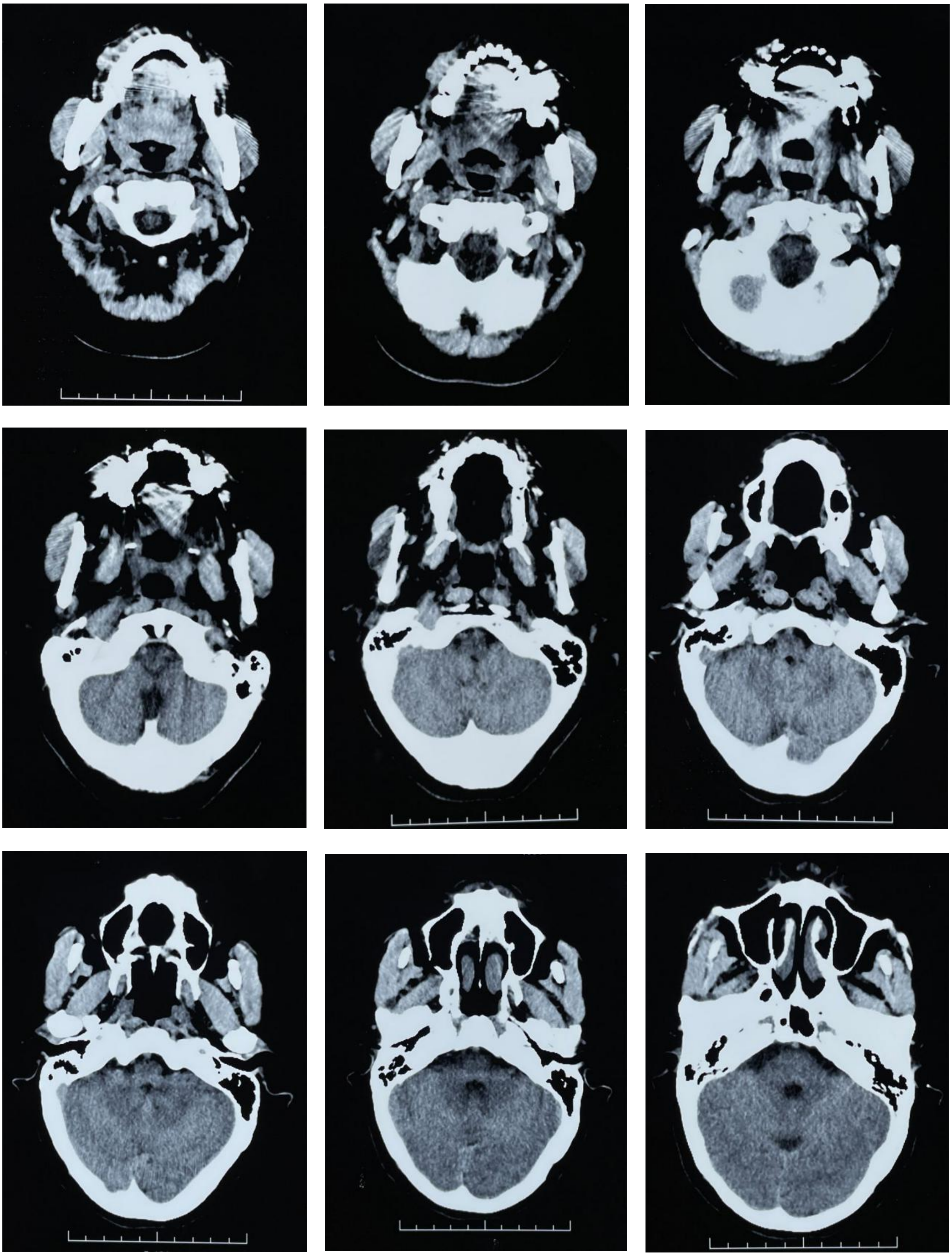

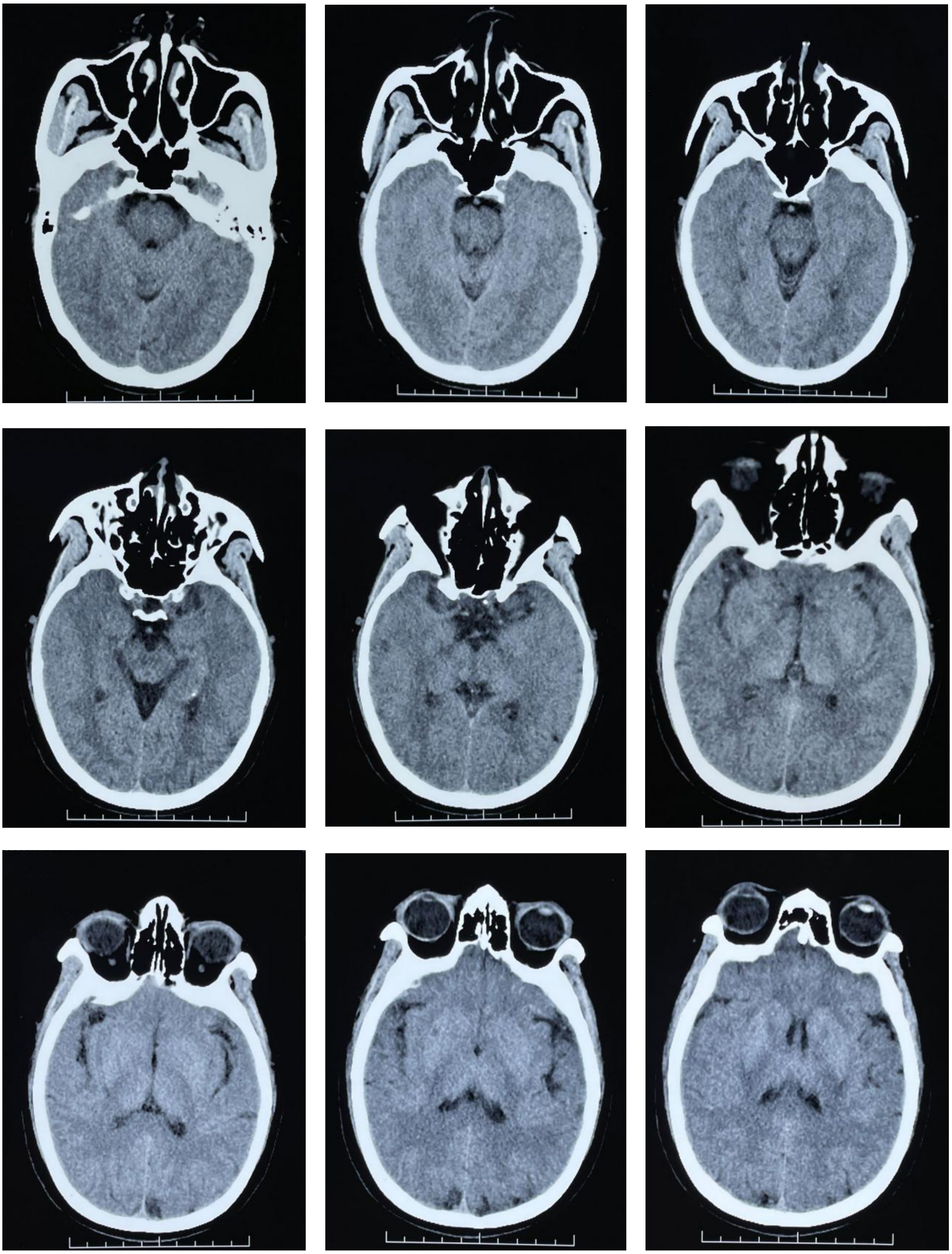

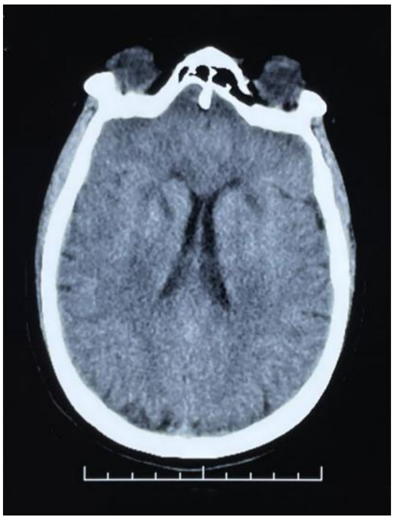

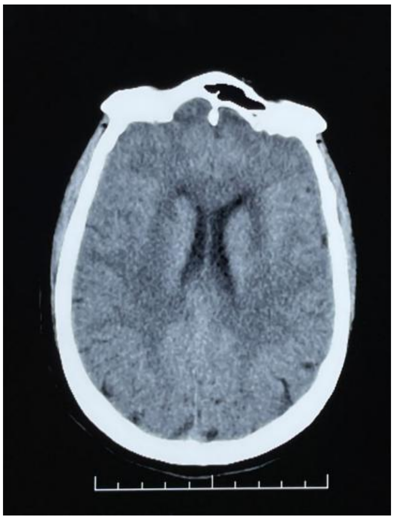

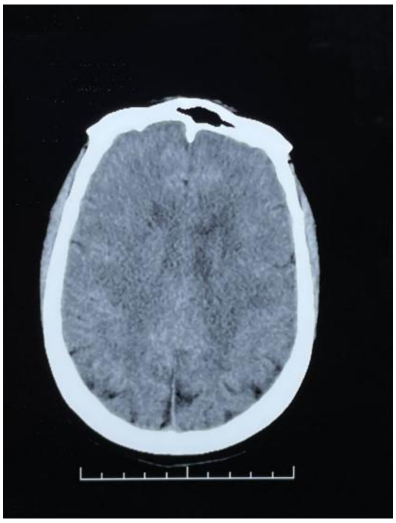

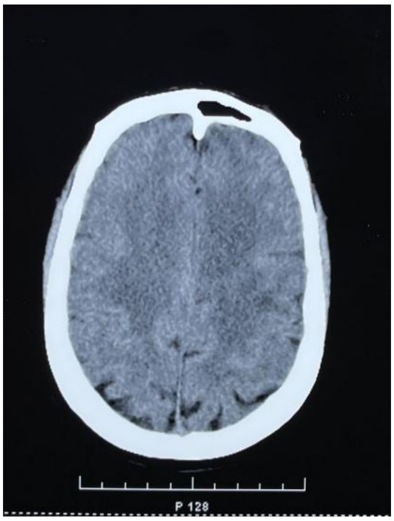
P 128

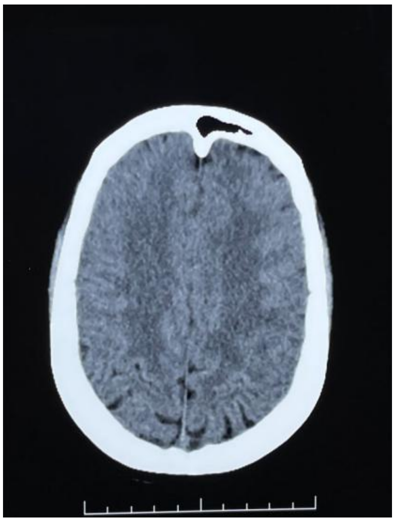

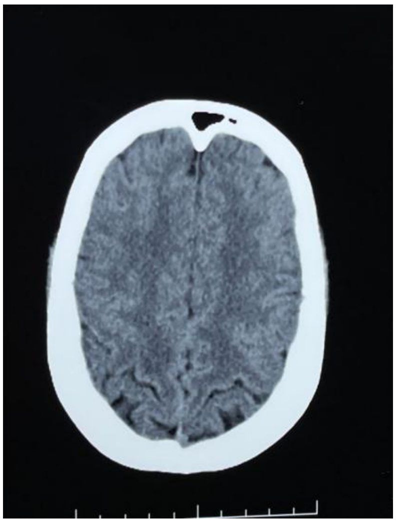

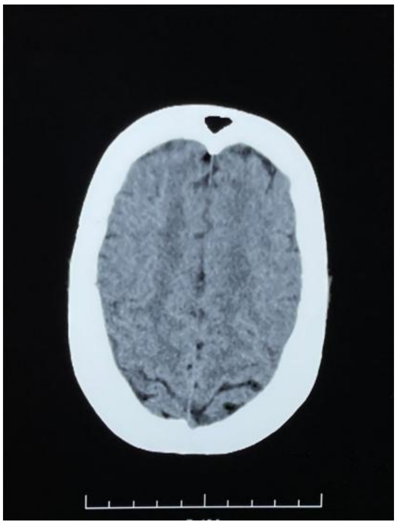

C. Brain CT scan images of individual II: 1

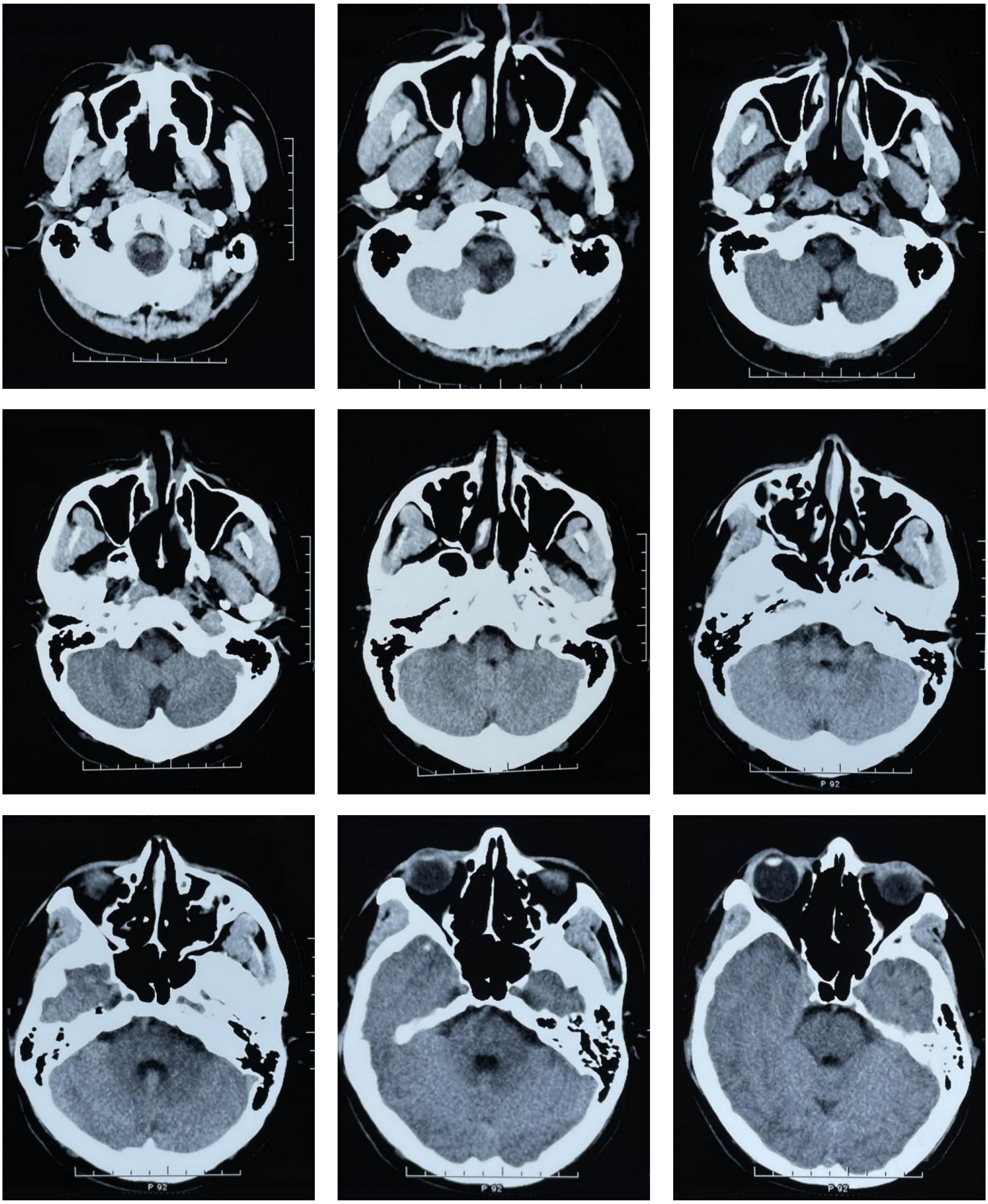

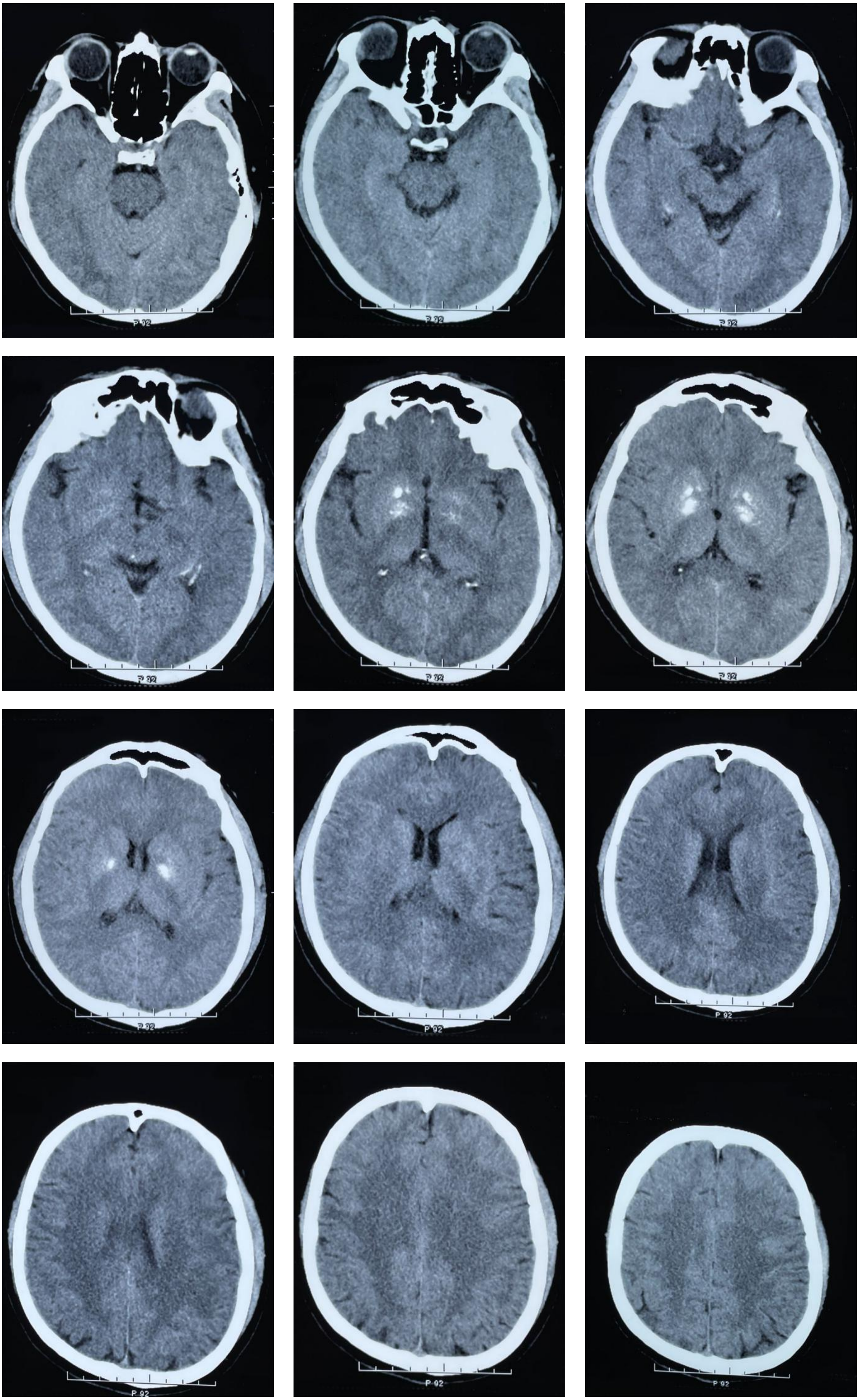

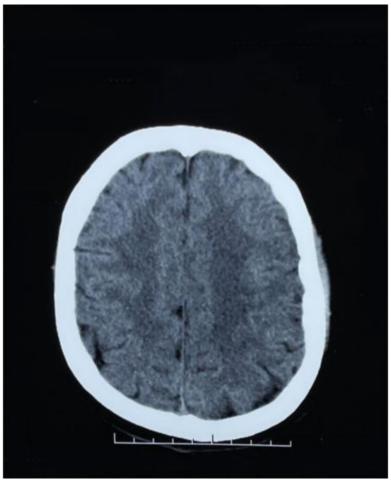
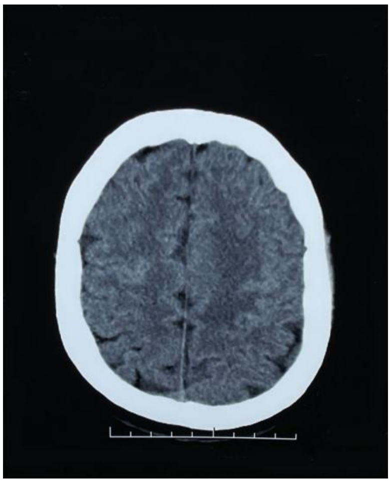
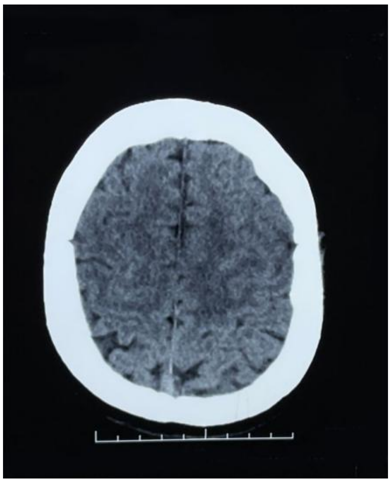
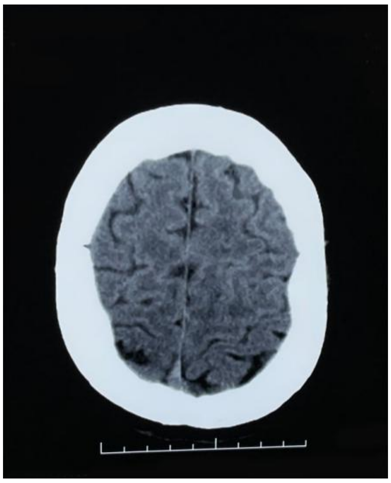

D. Brain CT scan images of individual II: 4

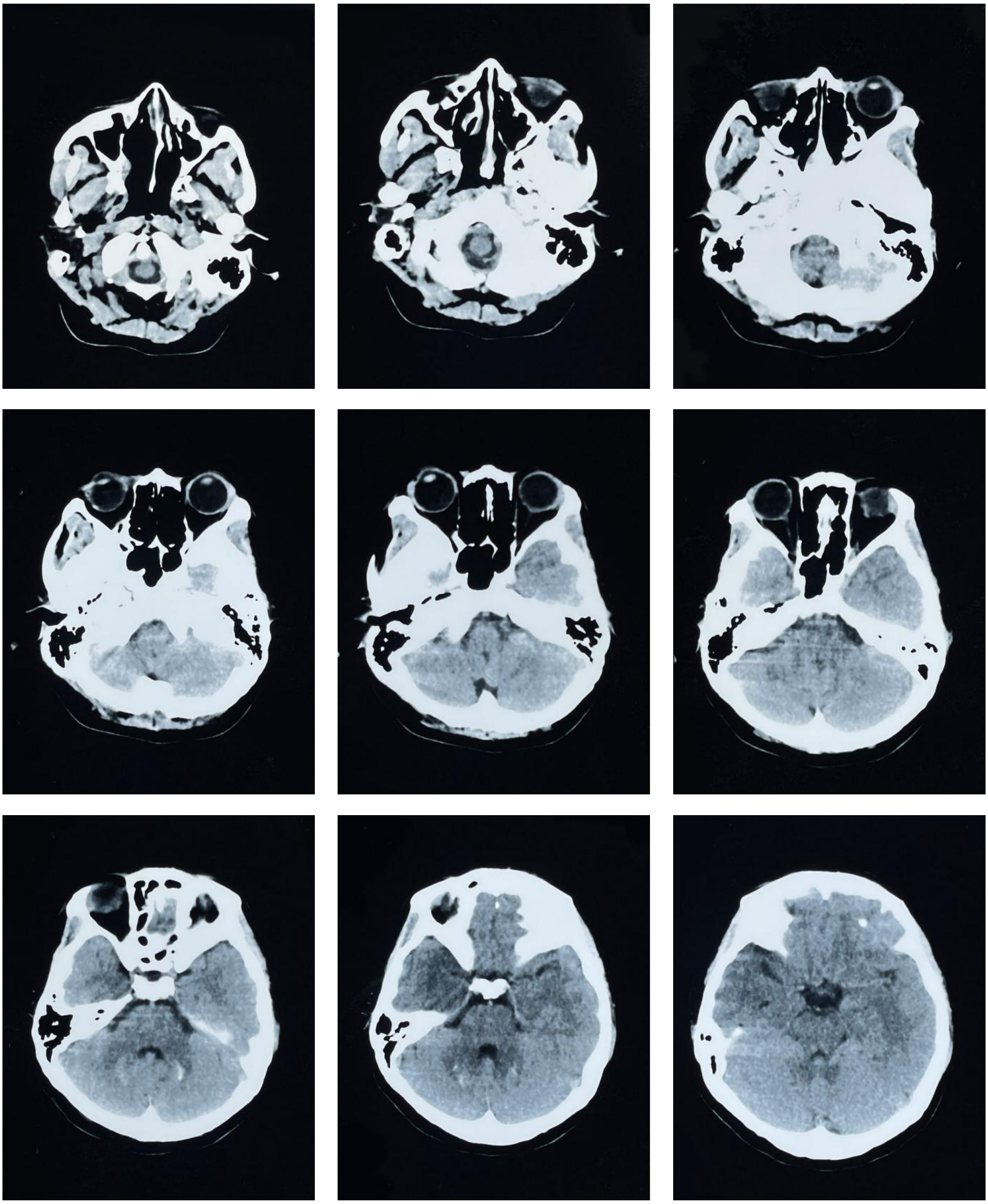

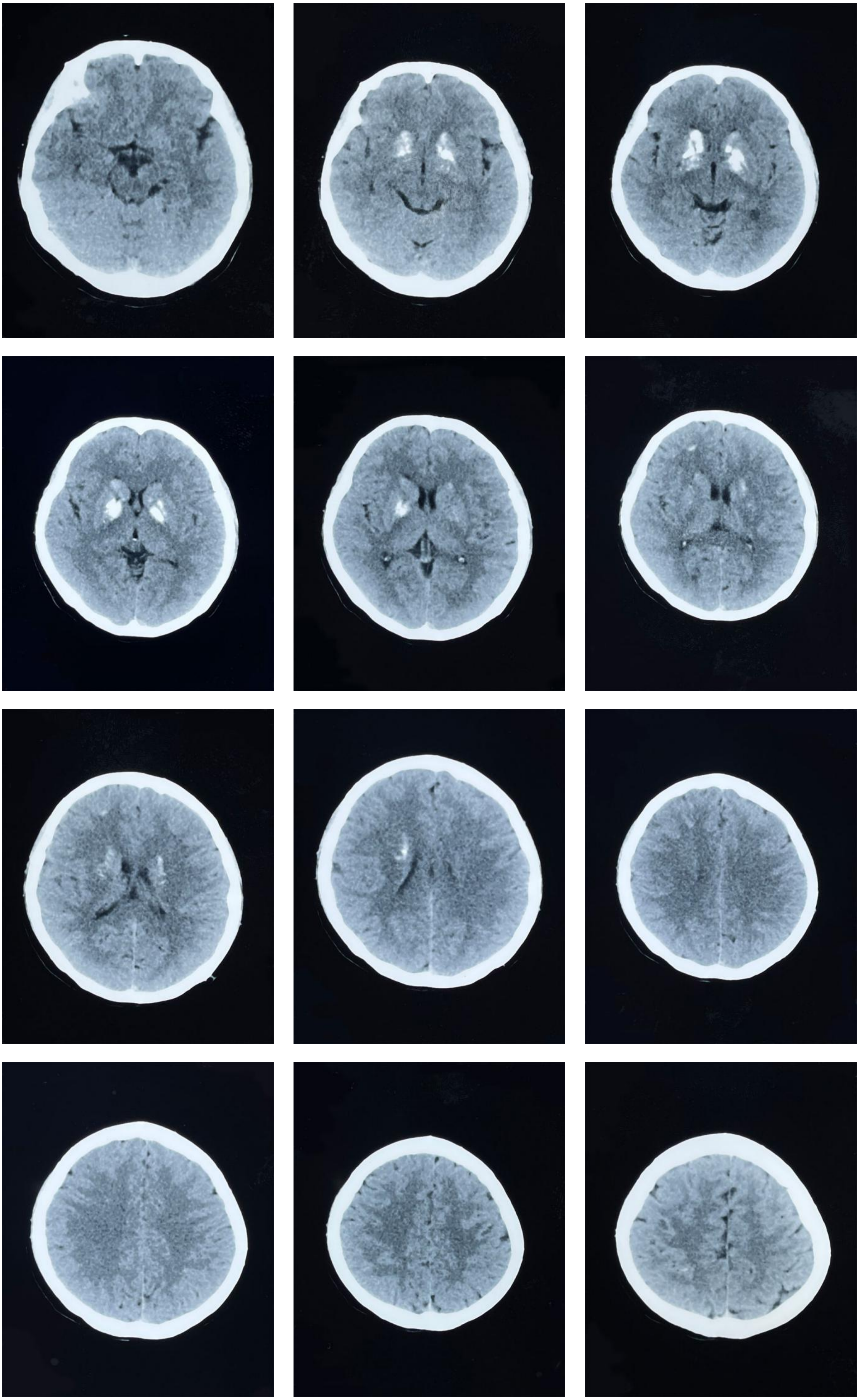

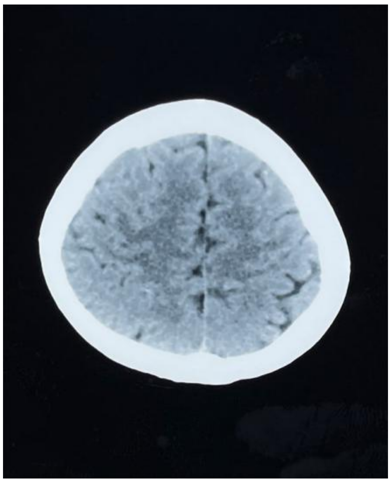
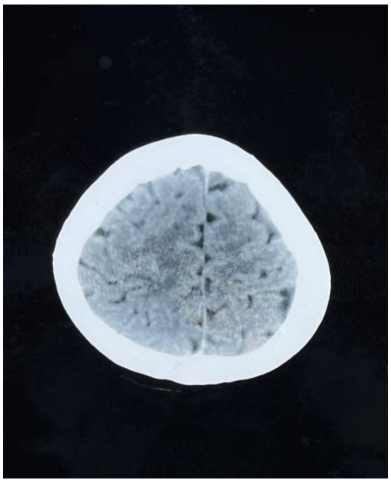
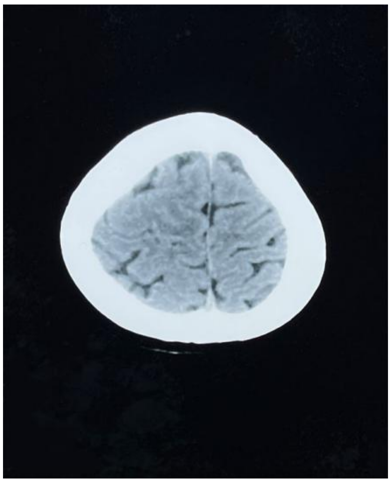
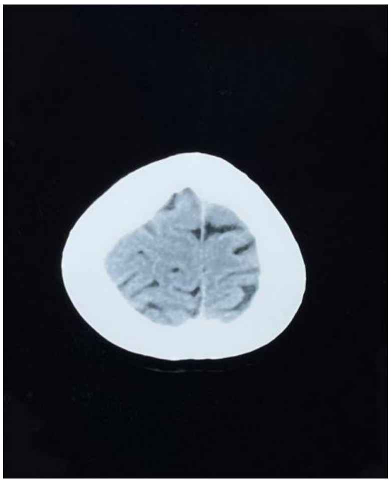

E. Brain CT scan images of individual III: 1

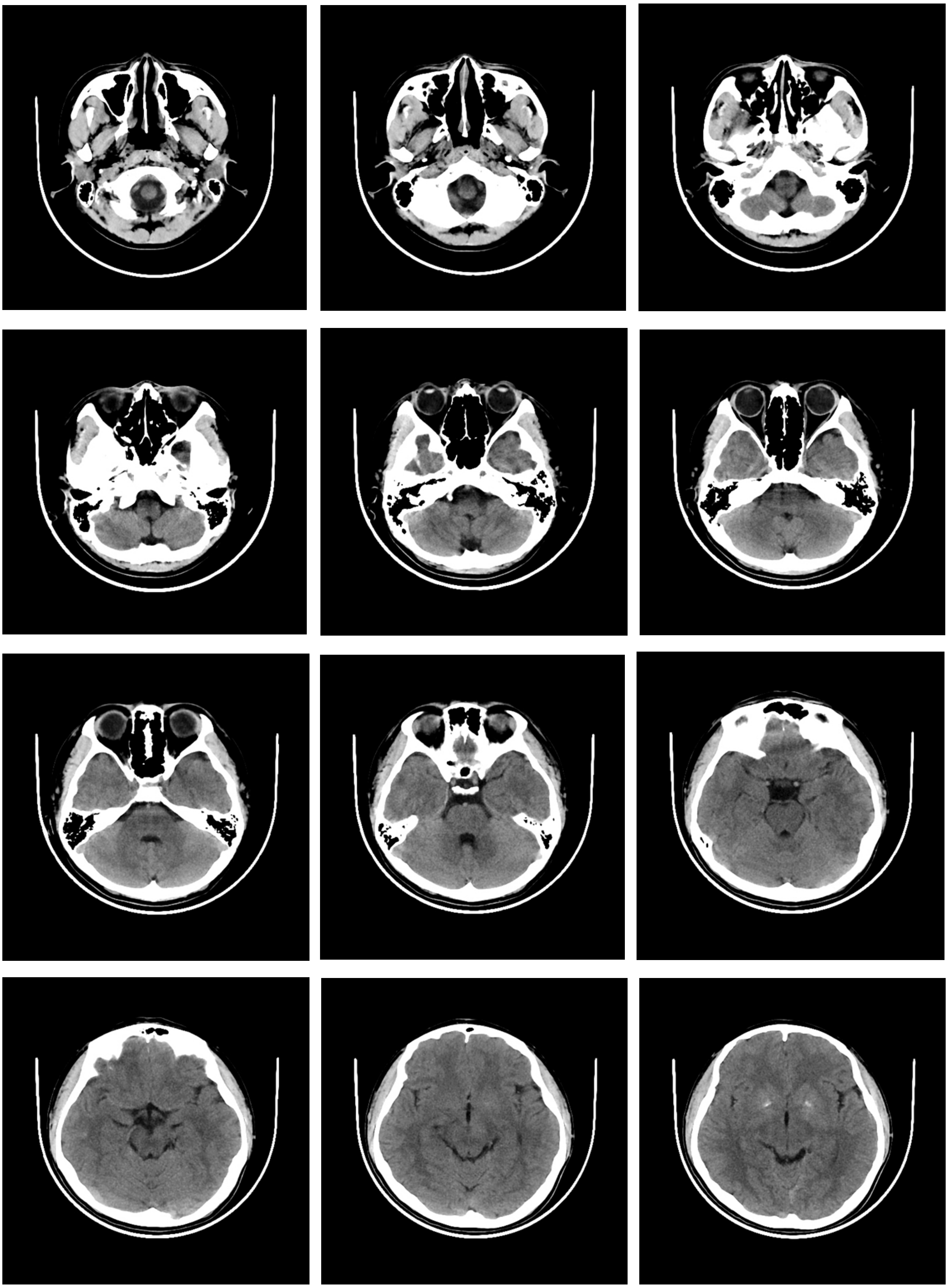

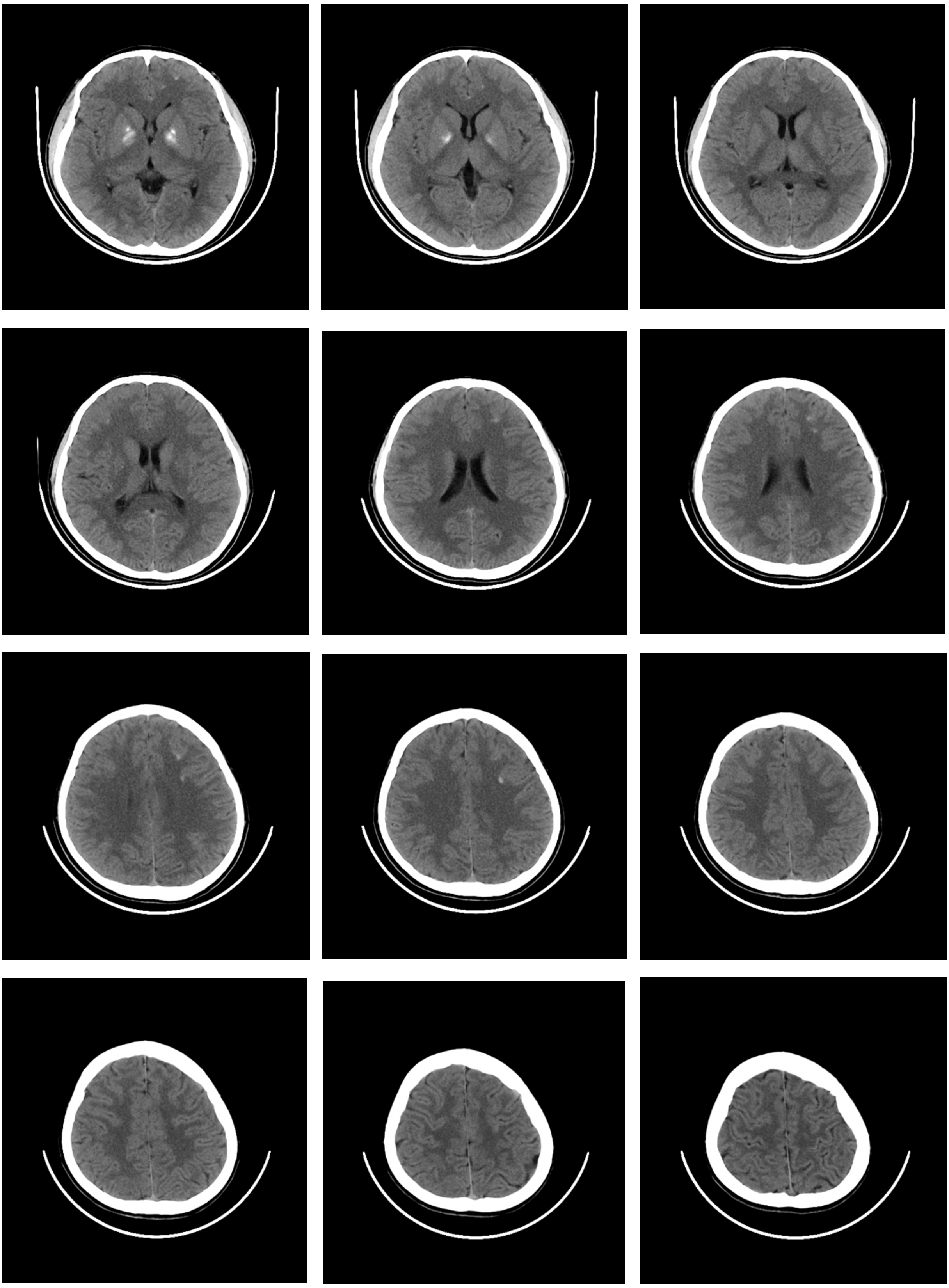

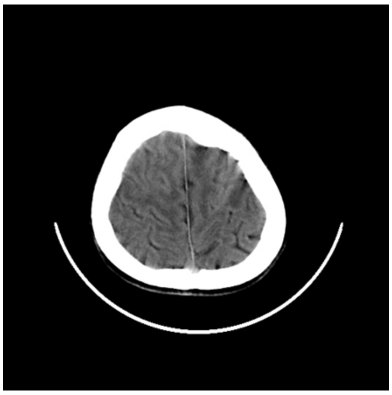
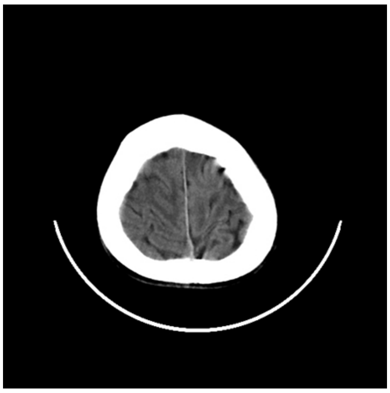
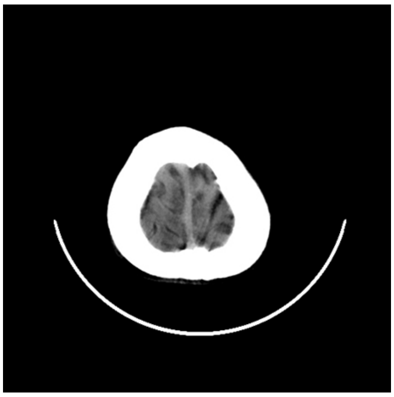
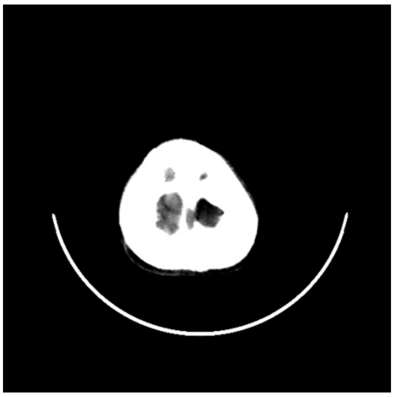

F. Brain CT scan images of individual III: 2

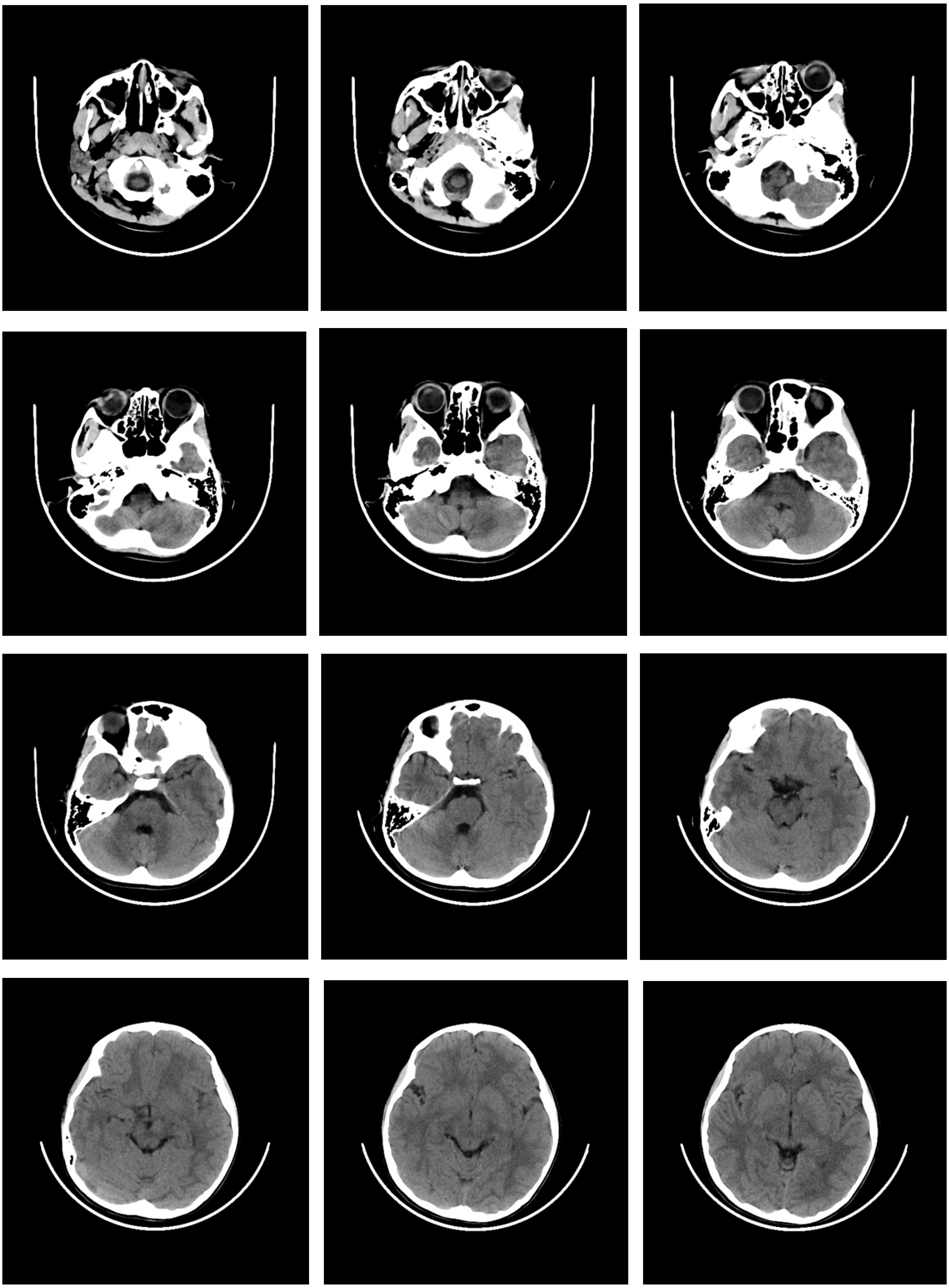

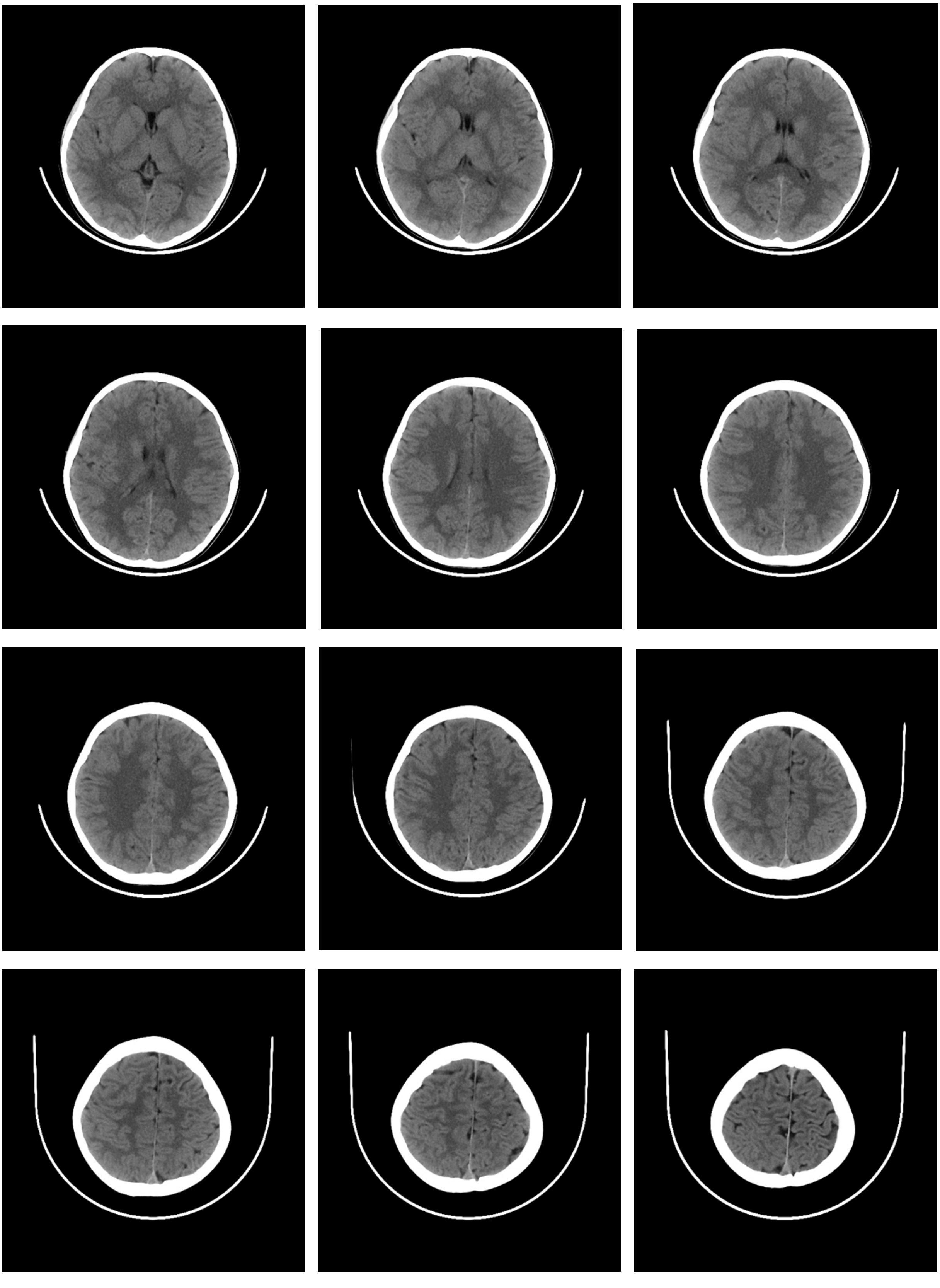

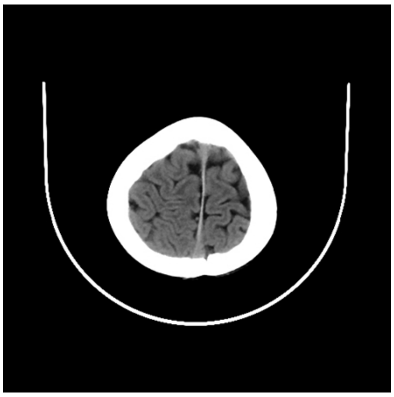
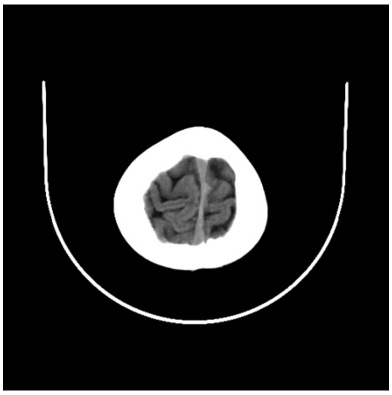
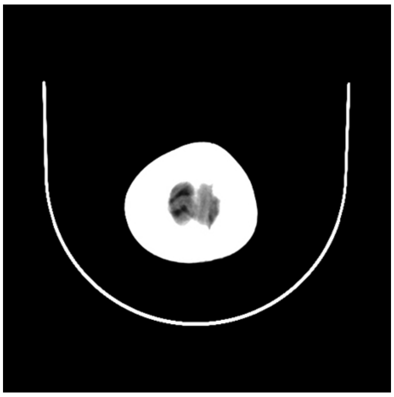
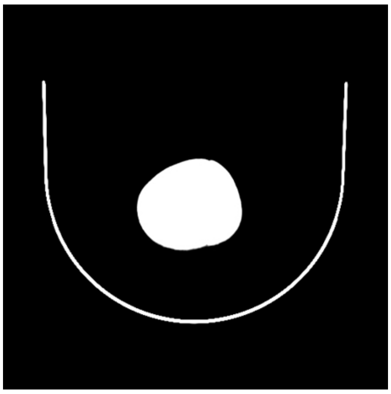

G. Brain CT scan images of individual III: 3

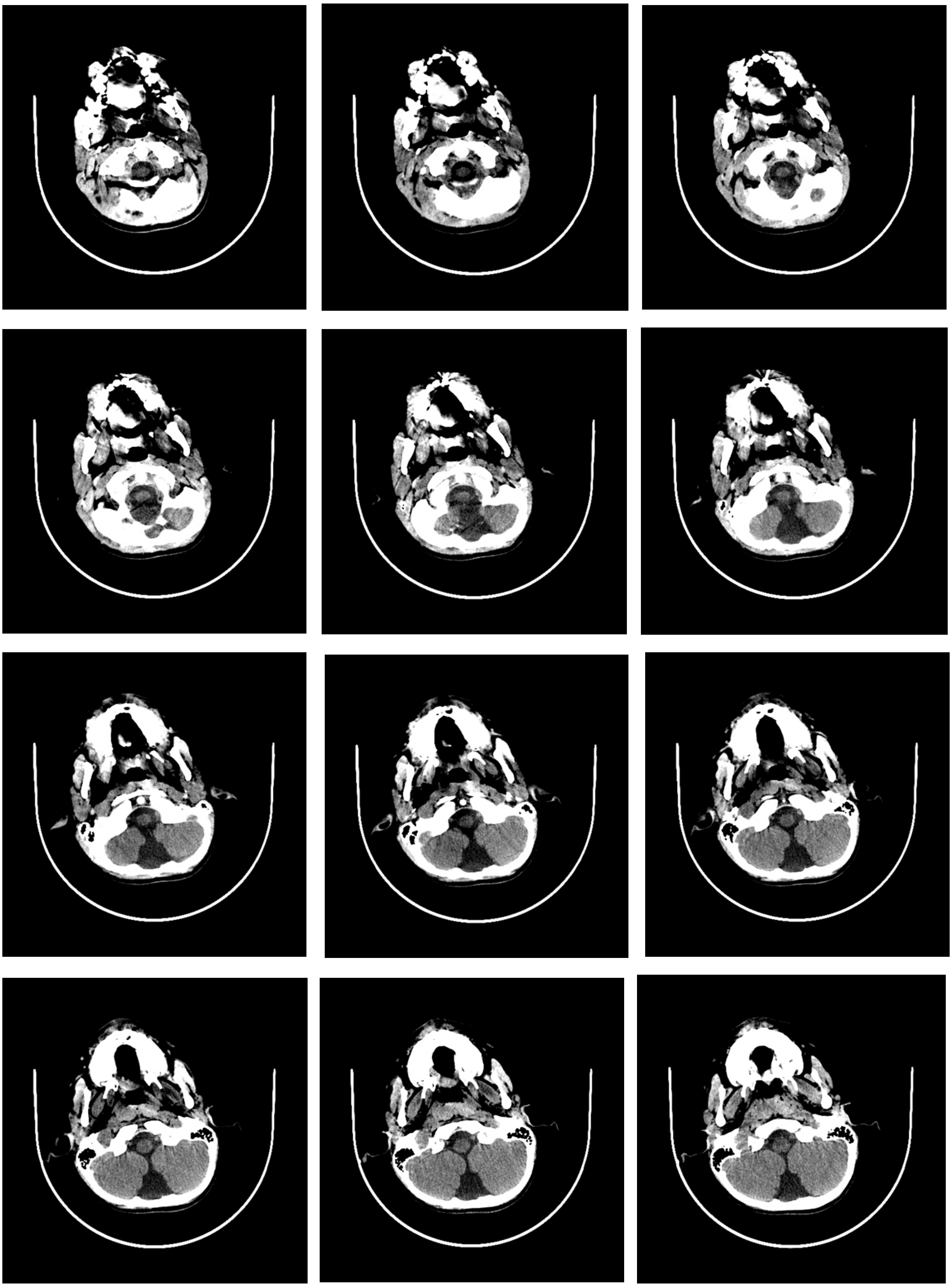

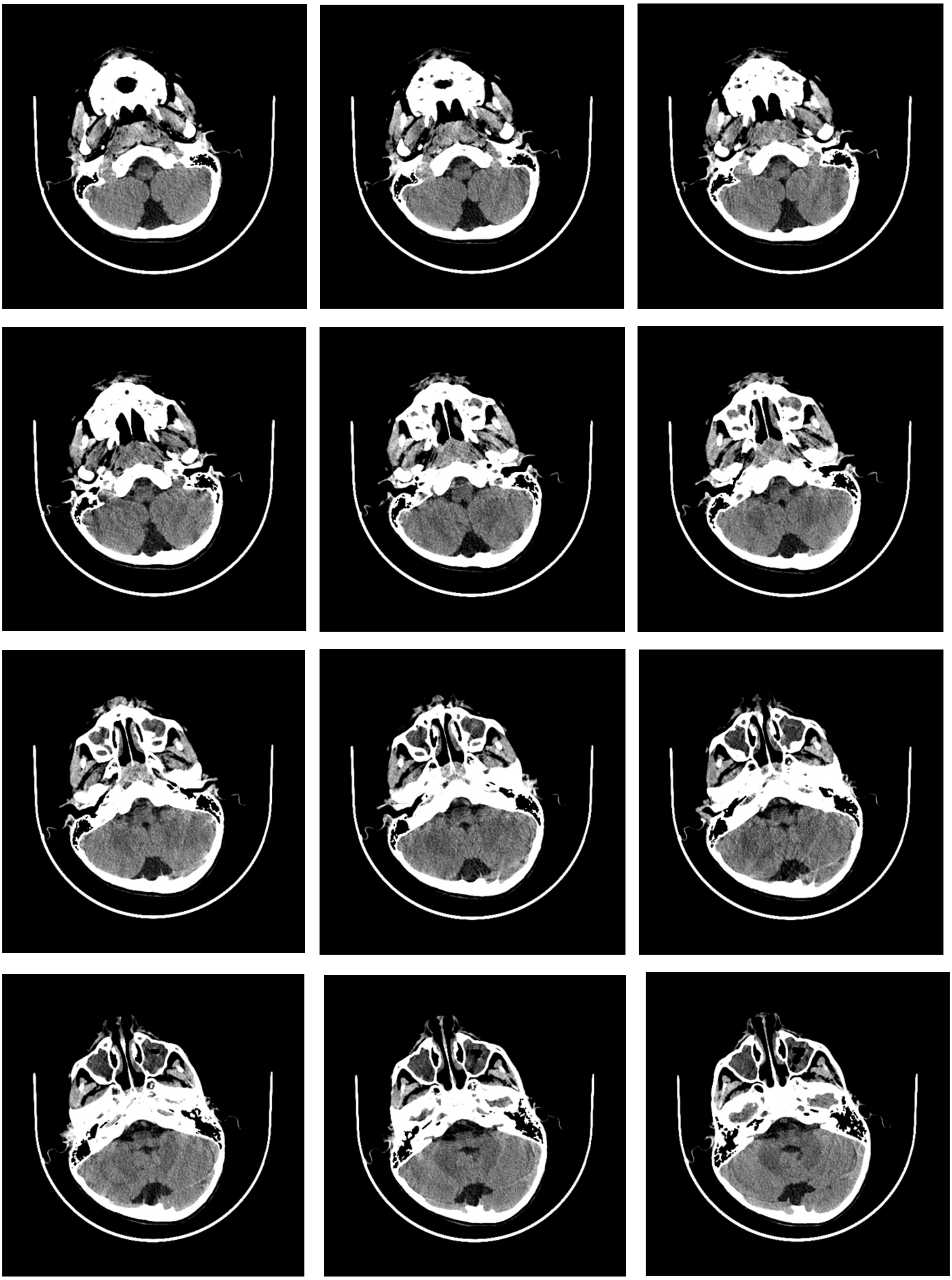

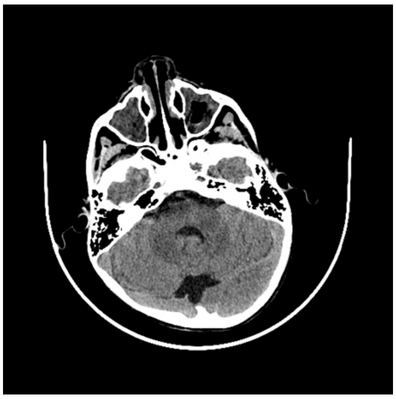
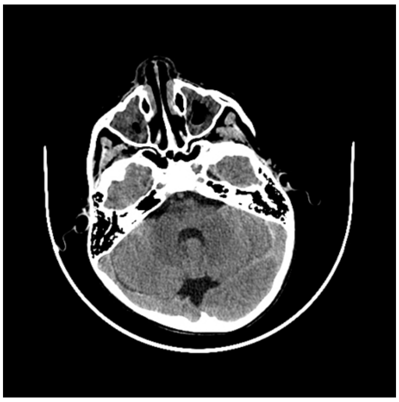
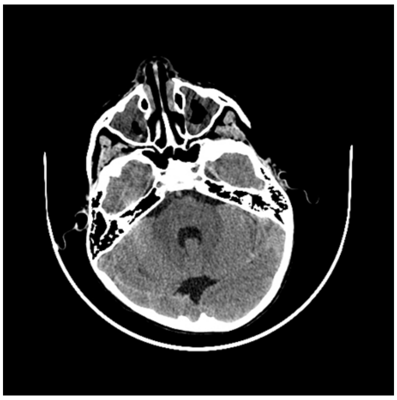
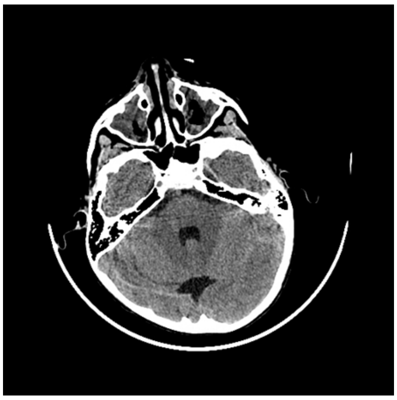
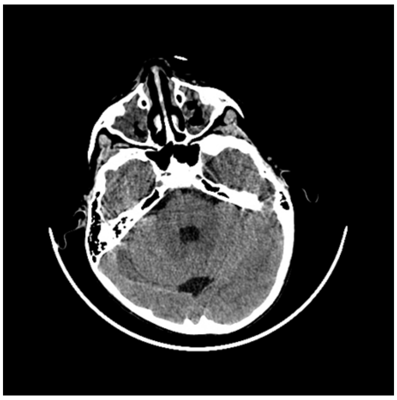
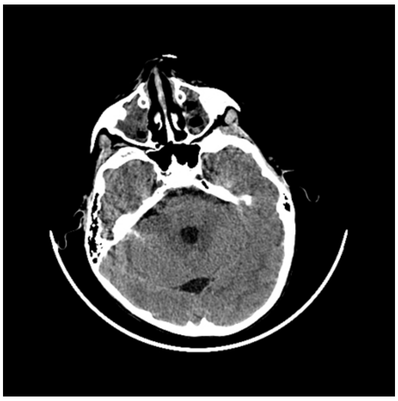
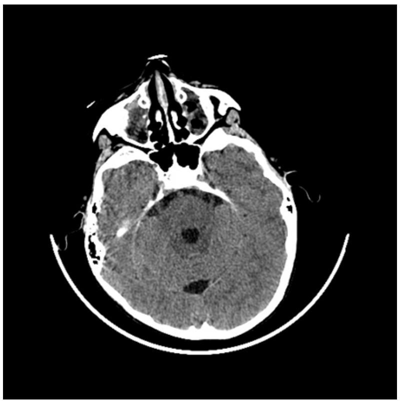
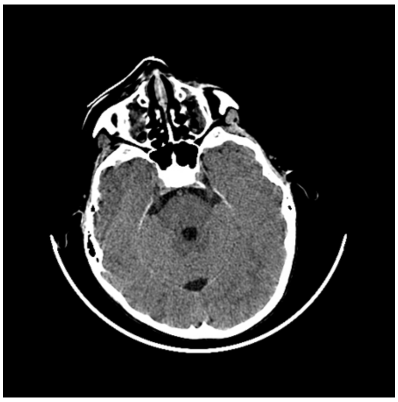
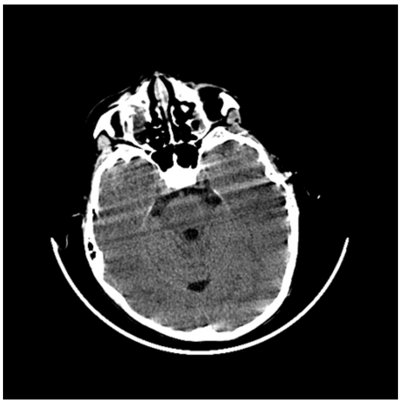
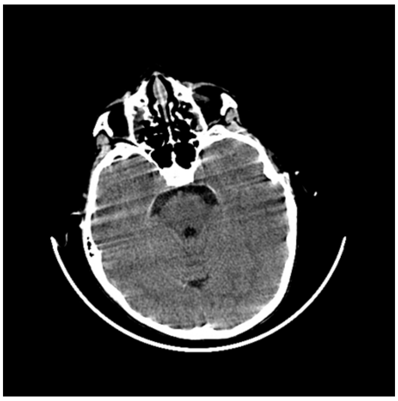
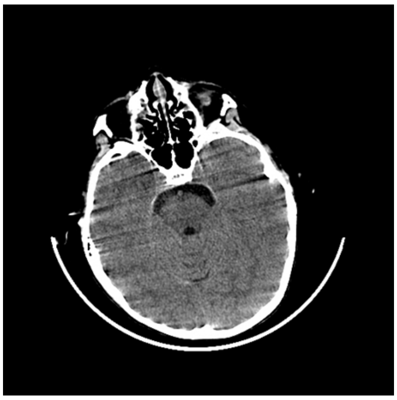
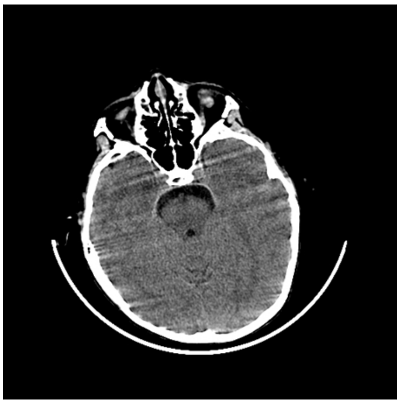

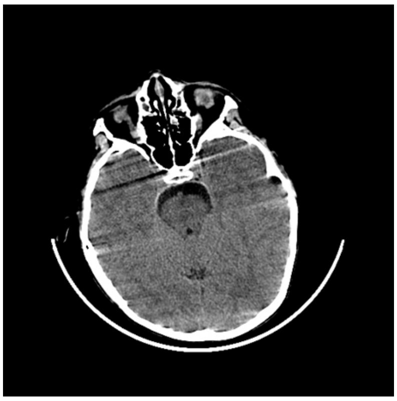
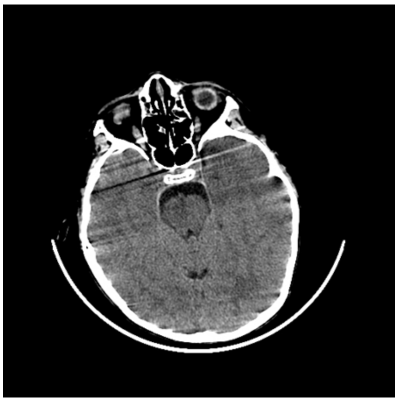
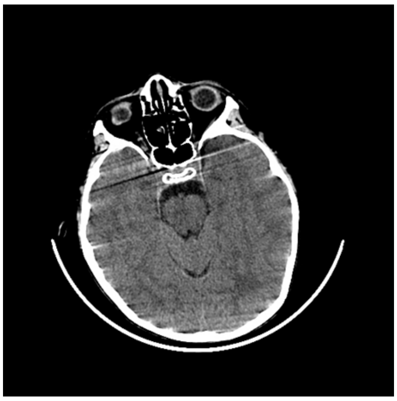
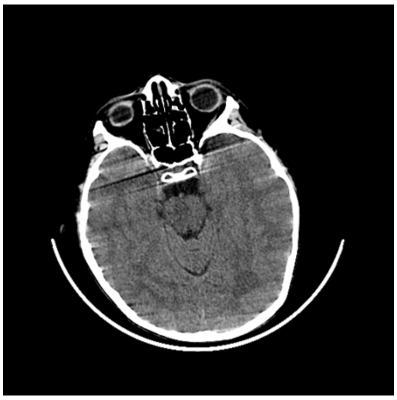
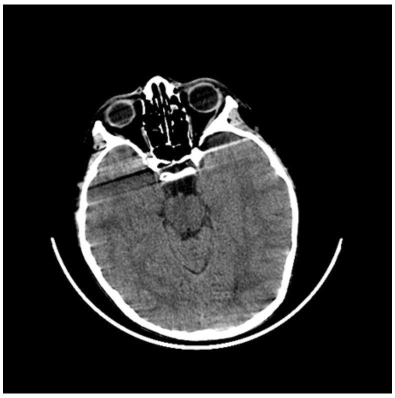
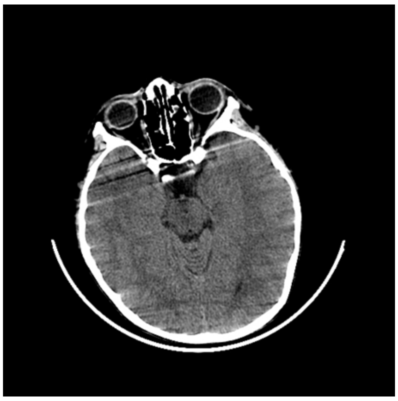
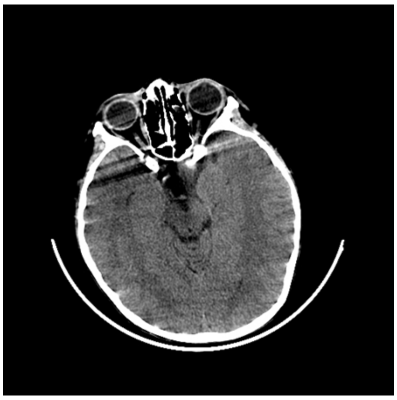
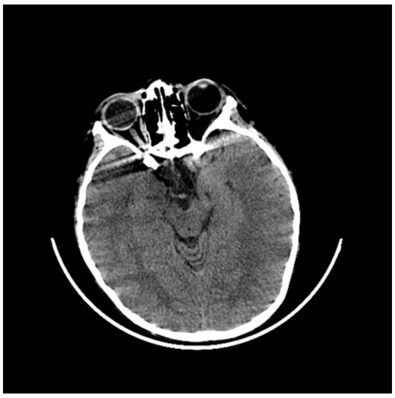
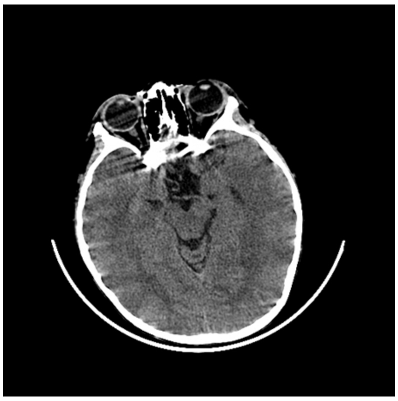
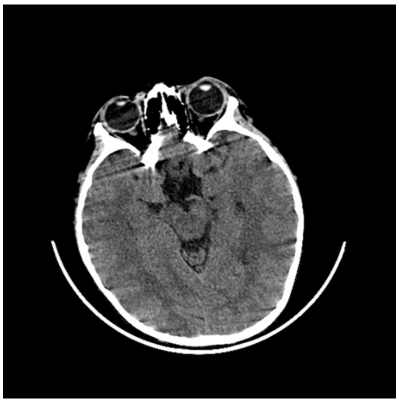
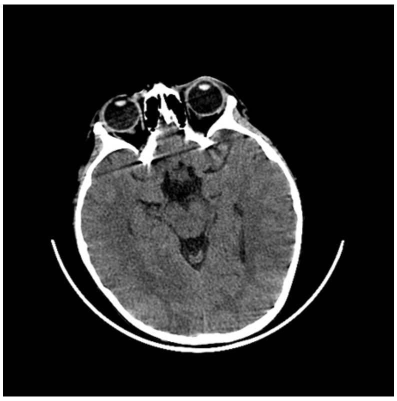
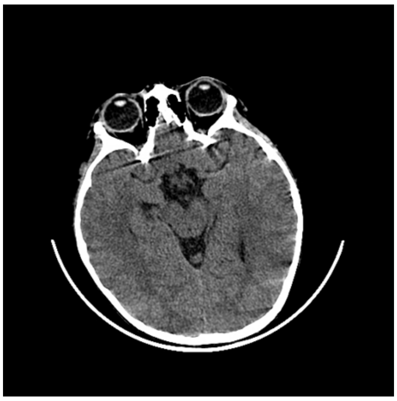

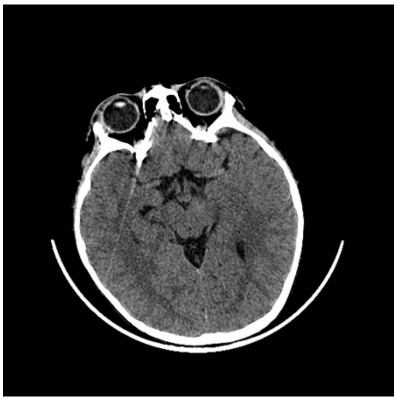
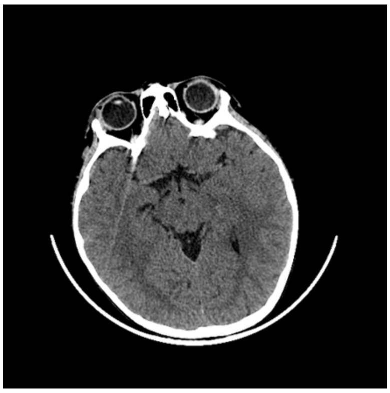
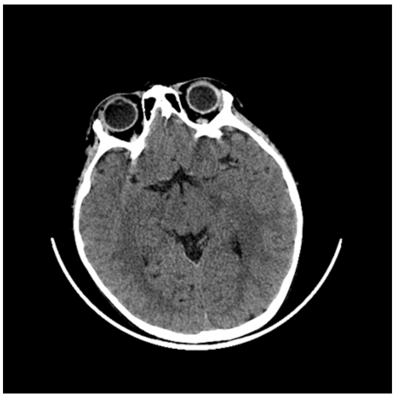
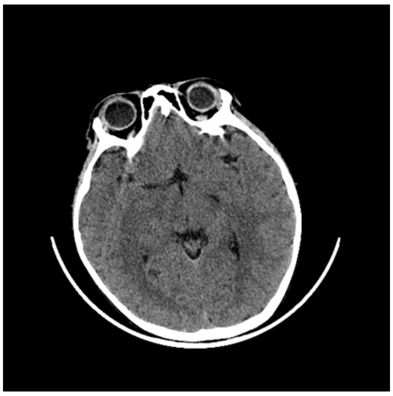
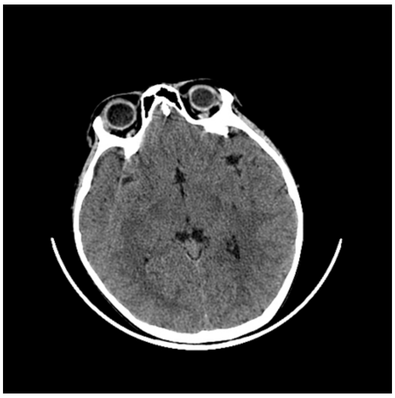
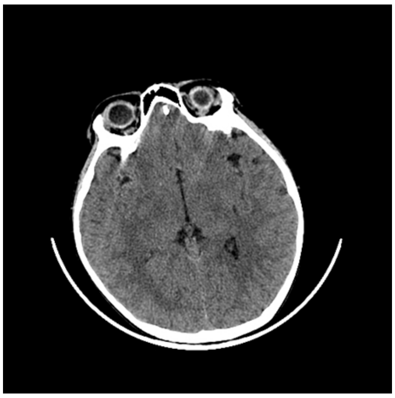
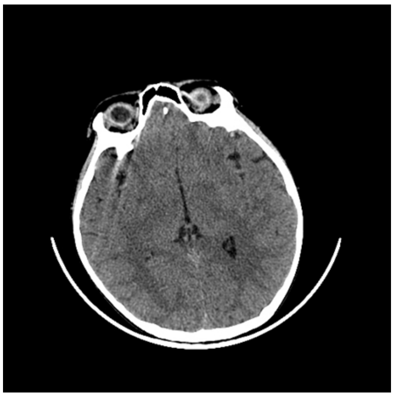
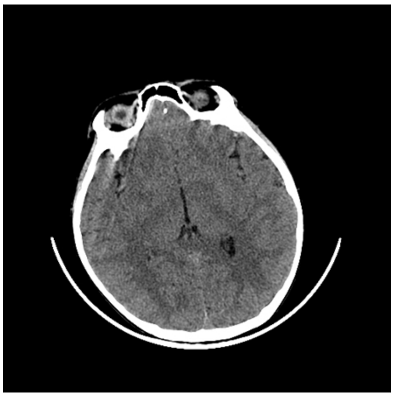
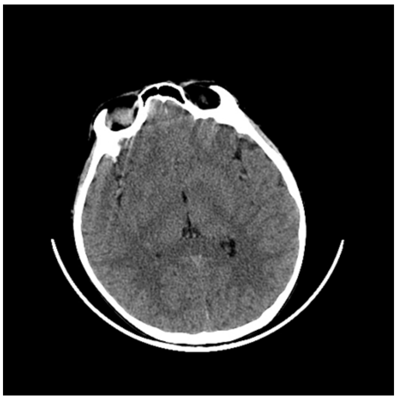
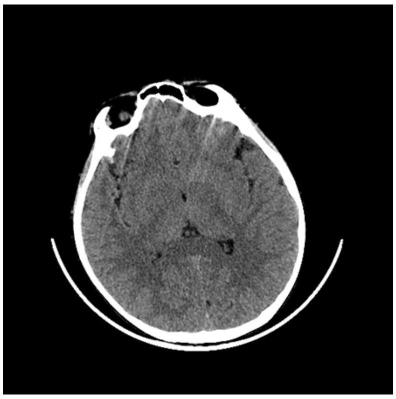
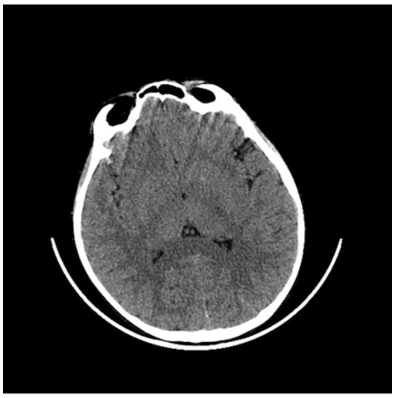
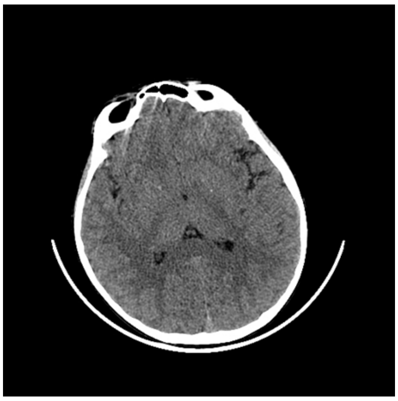

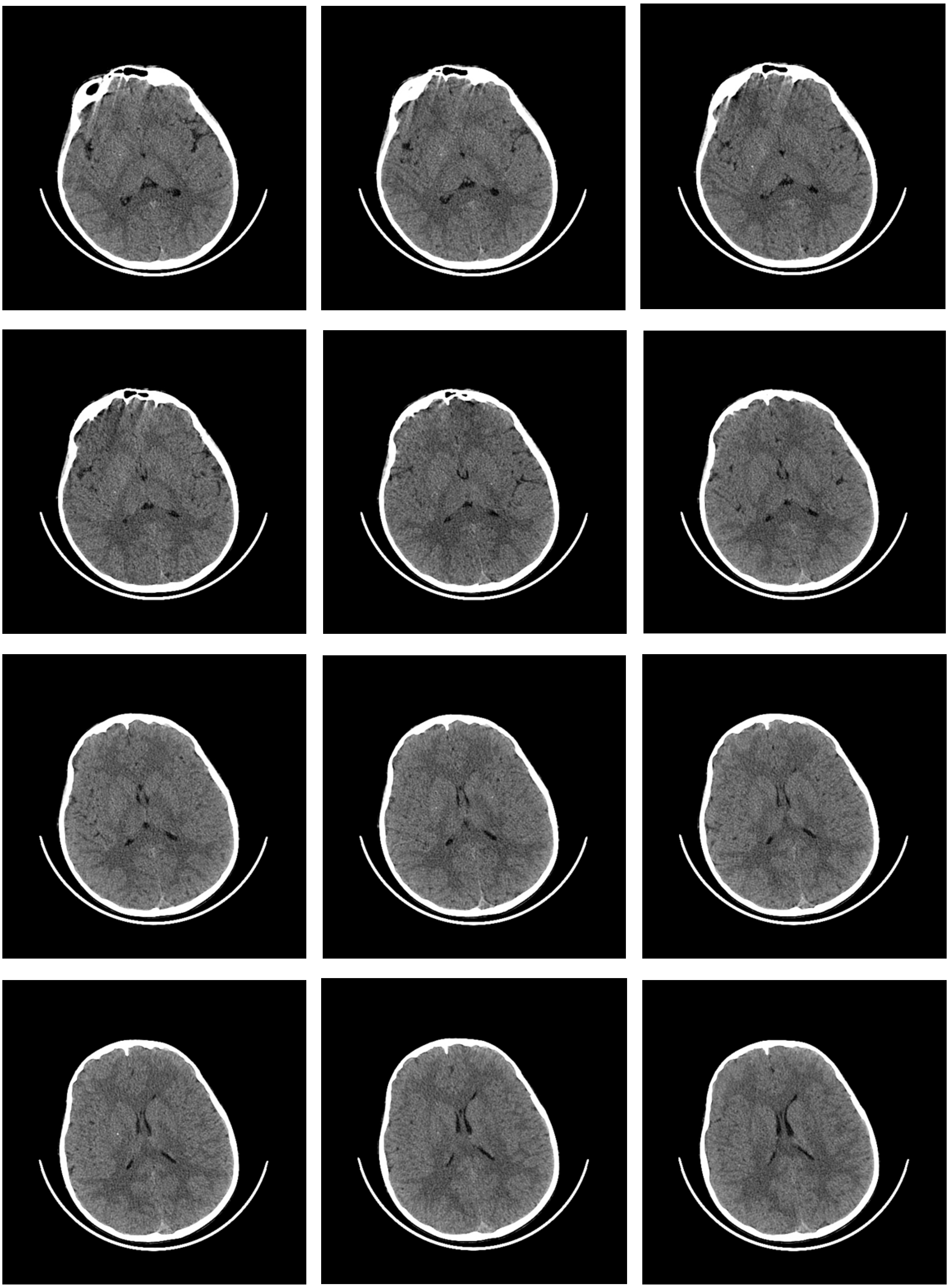

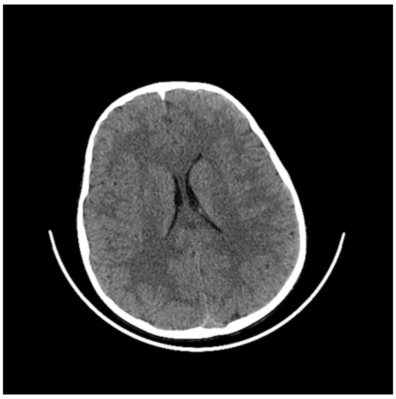
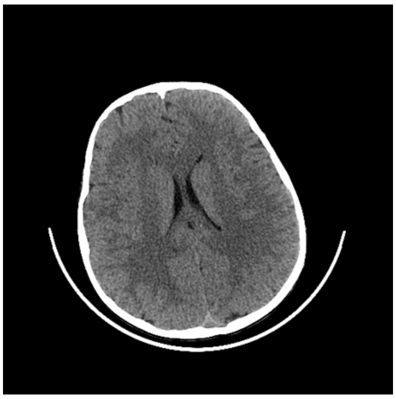
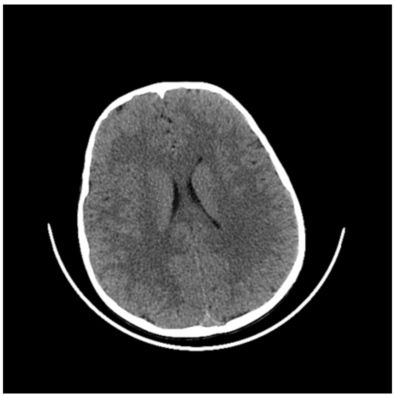
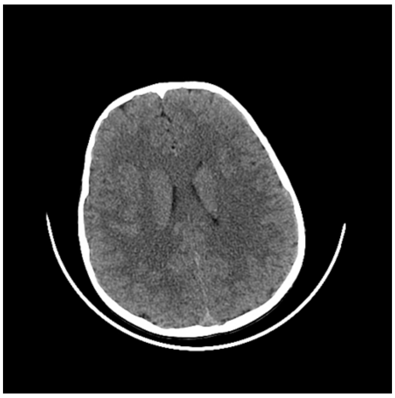
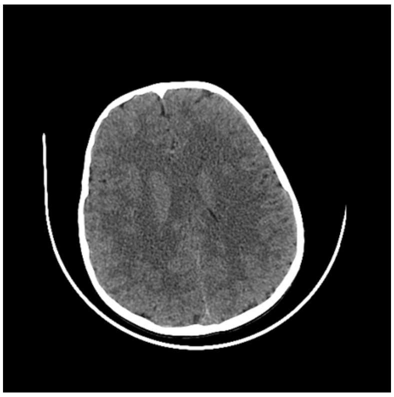
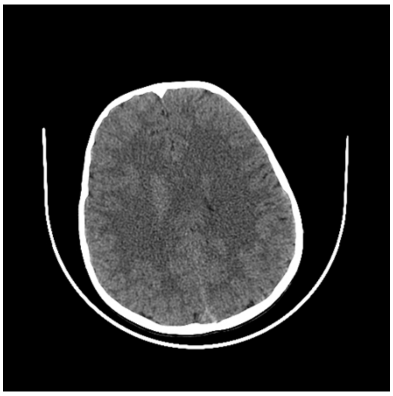
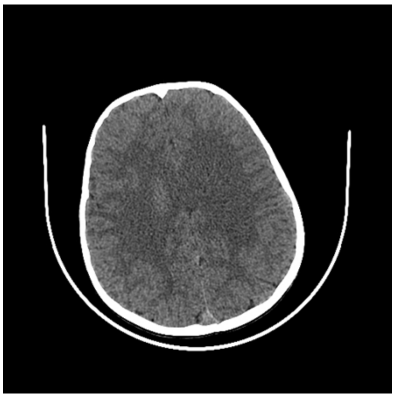
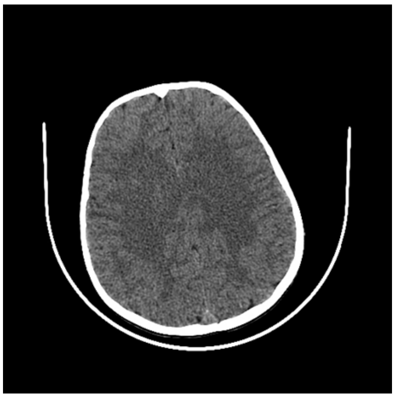
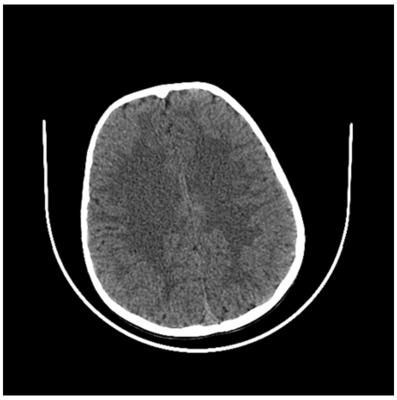
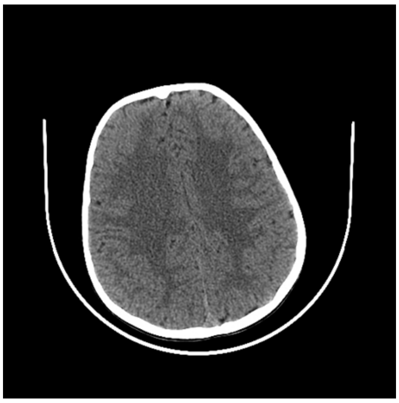
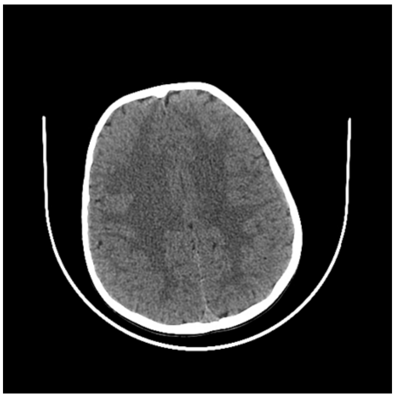
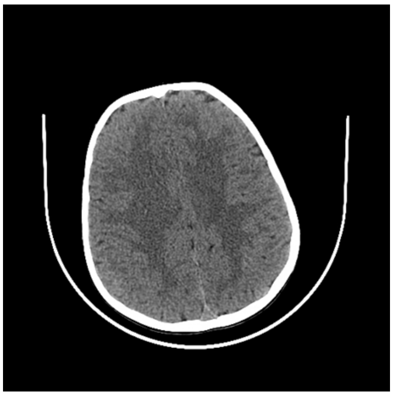

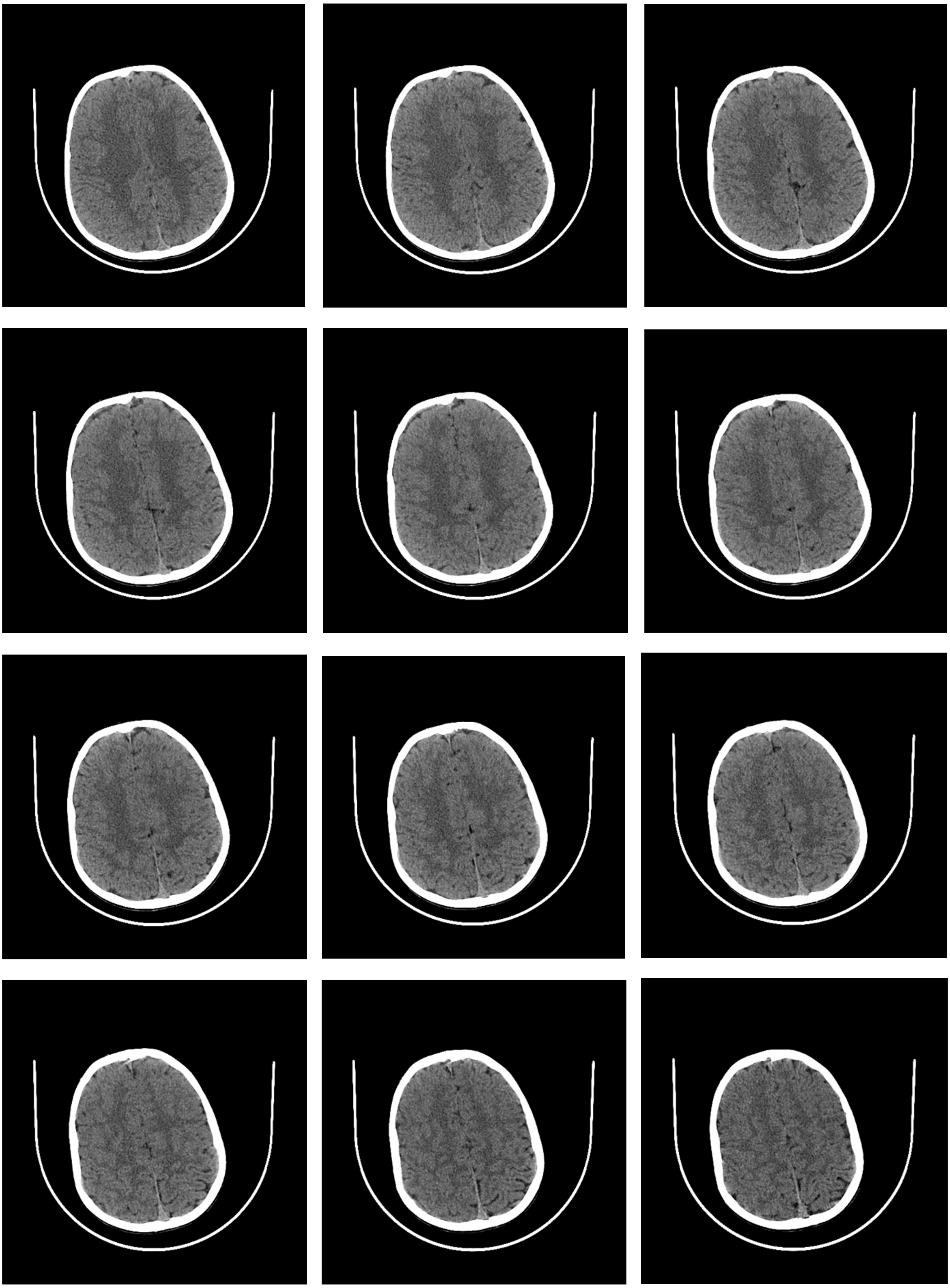

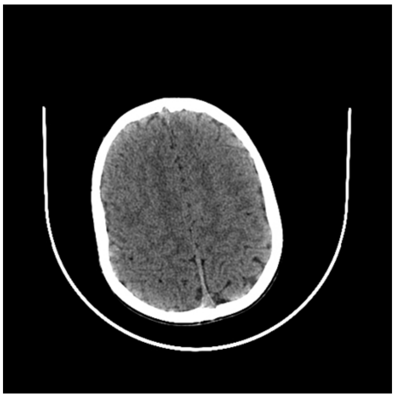
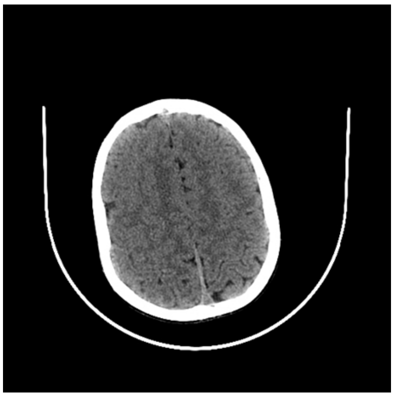
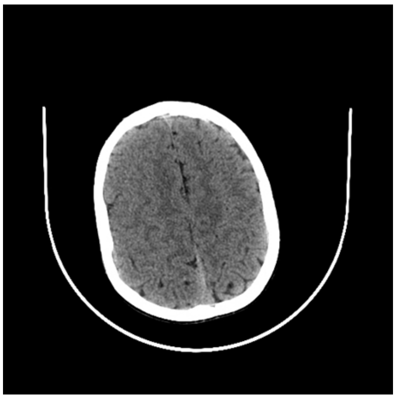
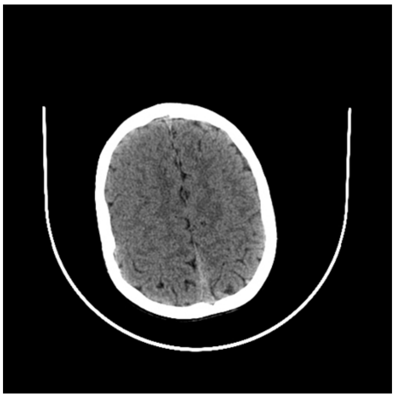
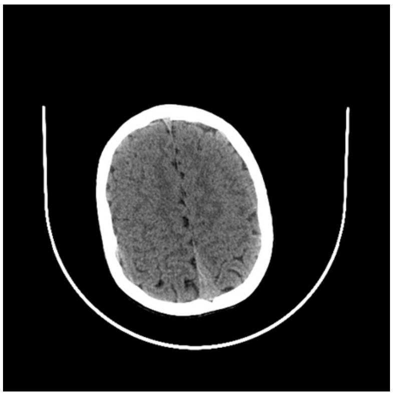
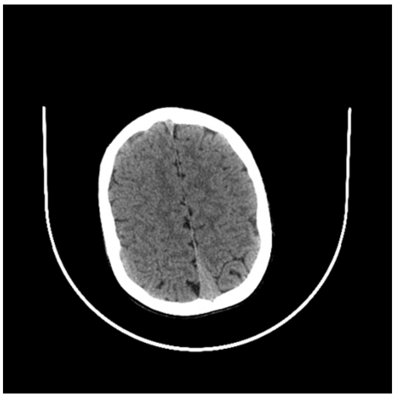
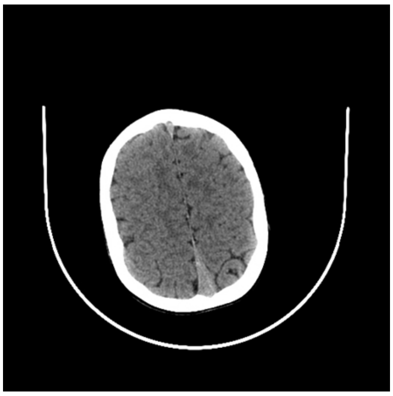
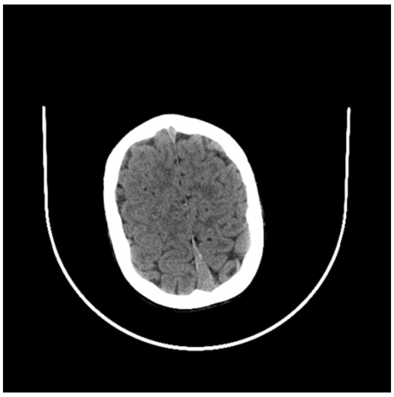
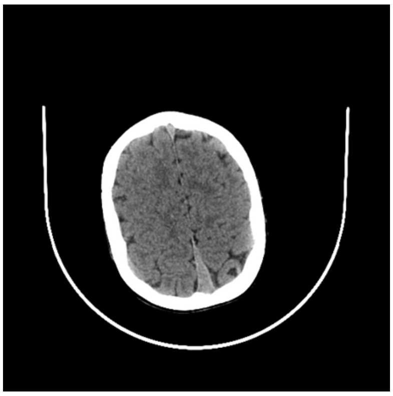
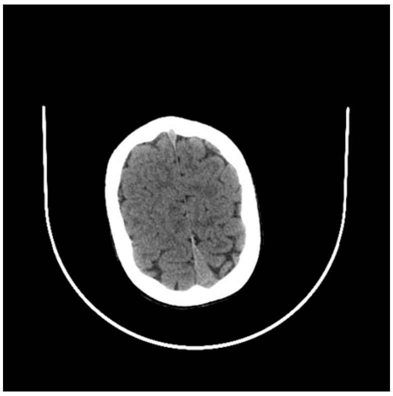
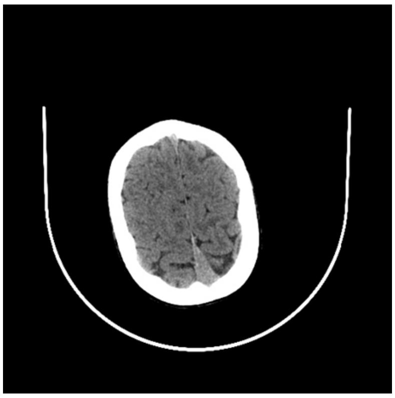
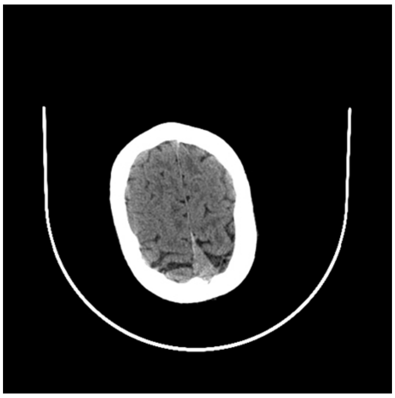

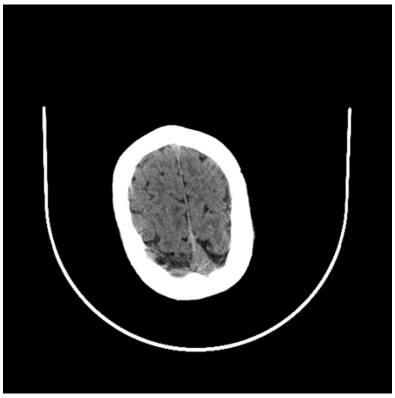
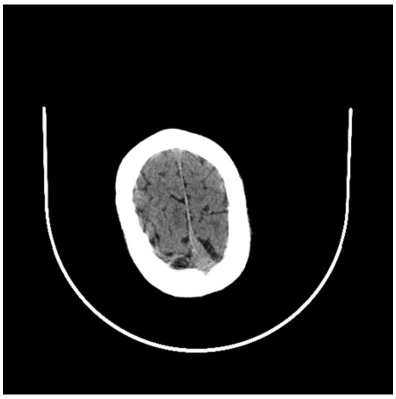
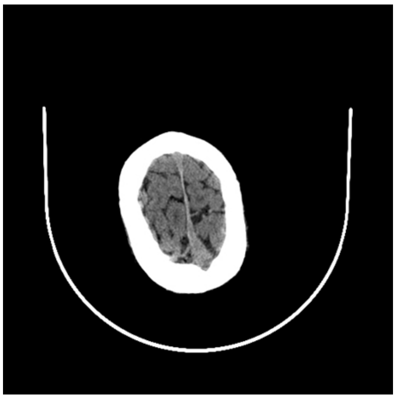
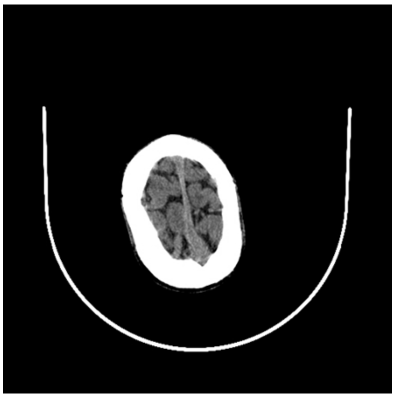

H. Brain CT scan images of individual III: 4

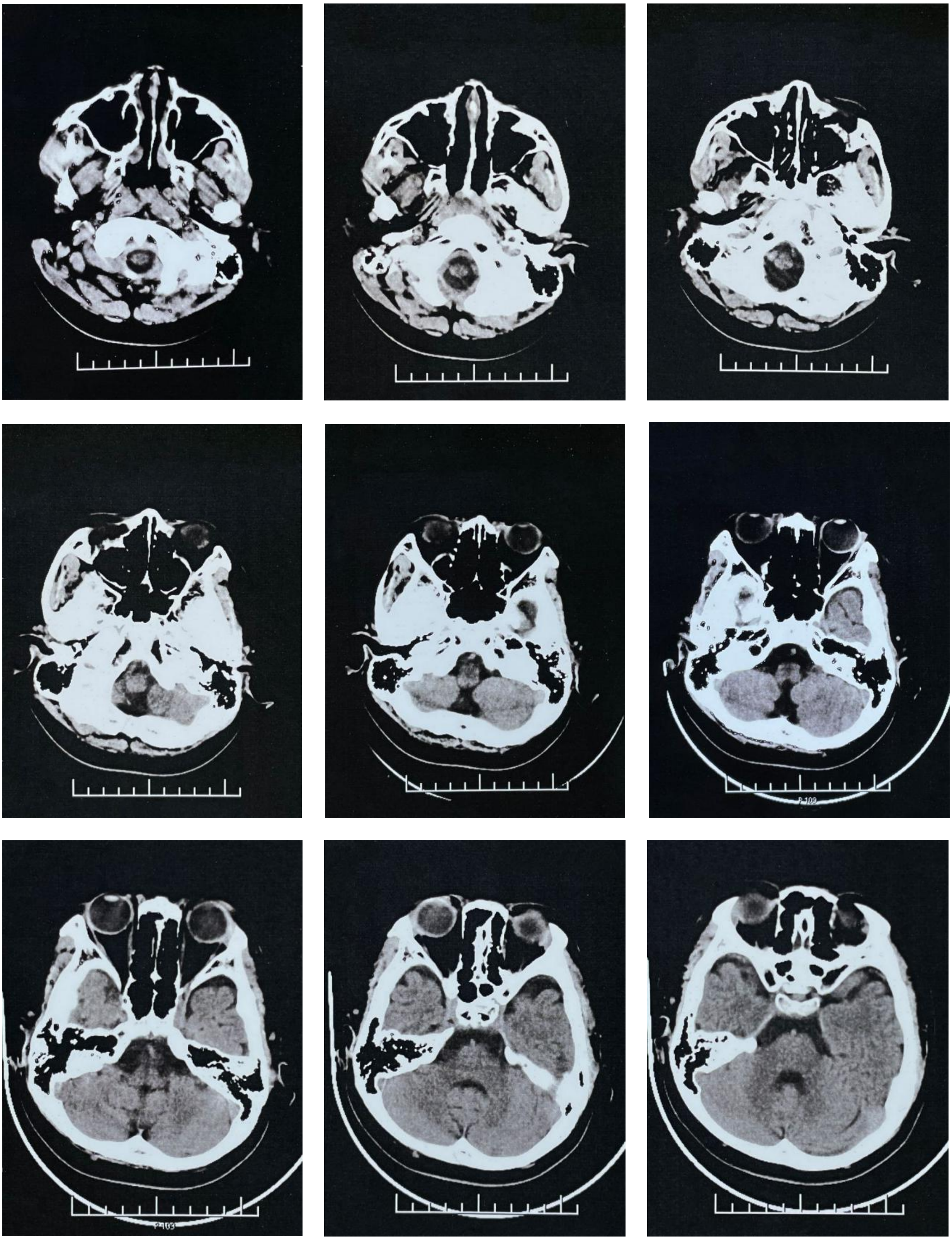

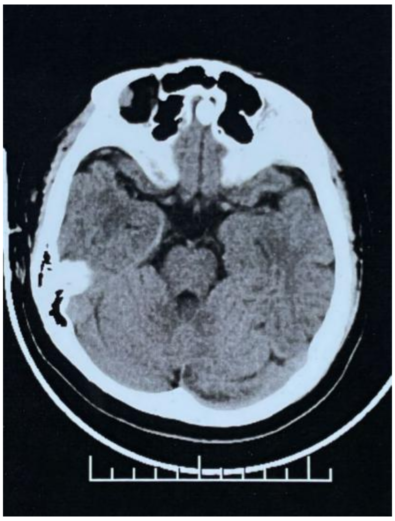
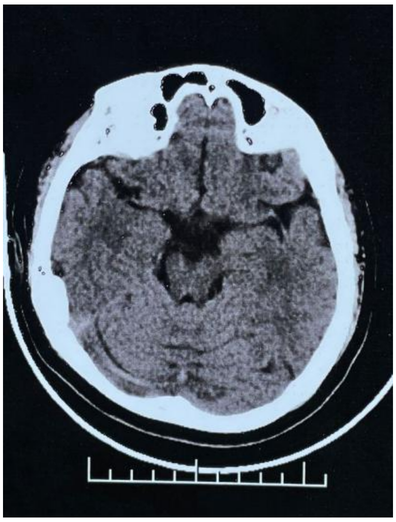
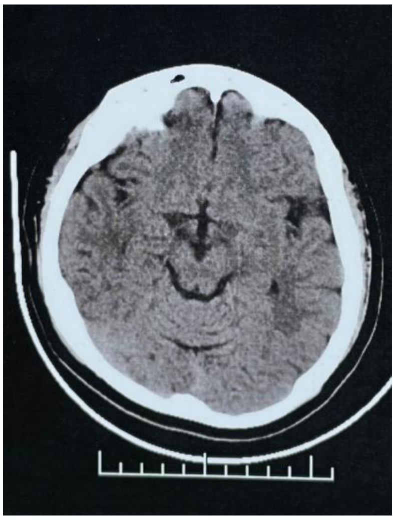
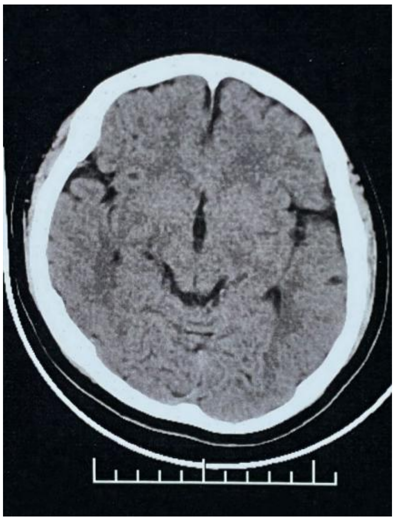
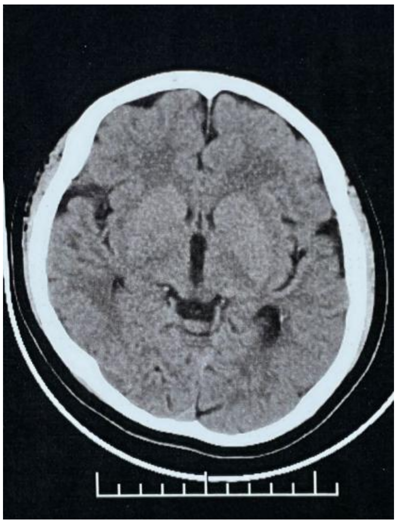
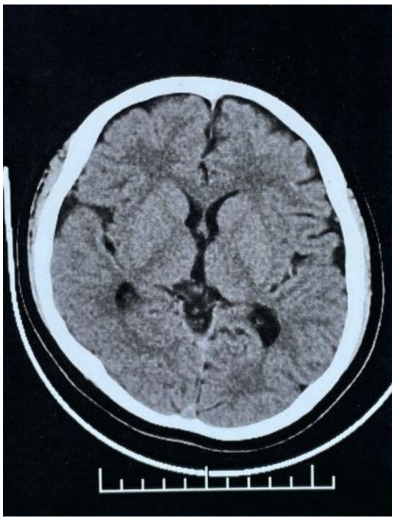
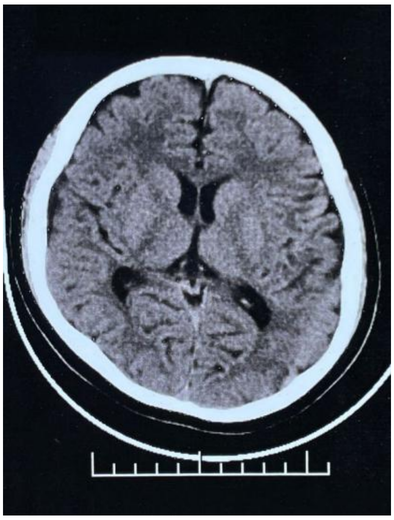
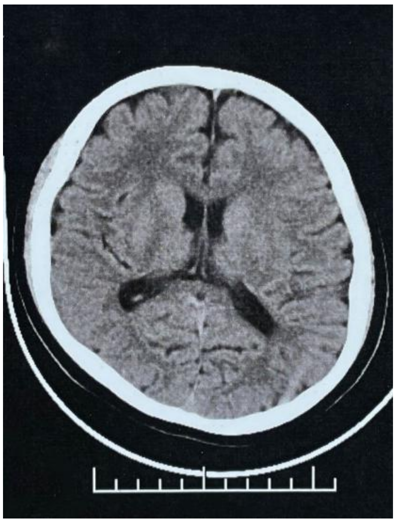
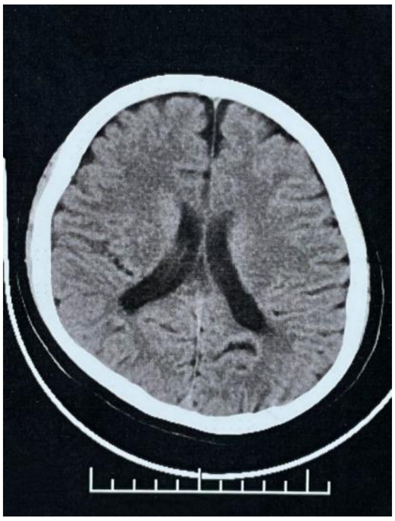

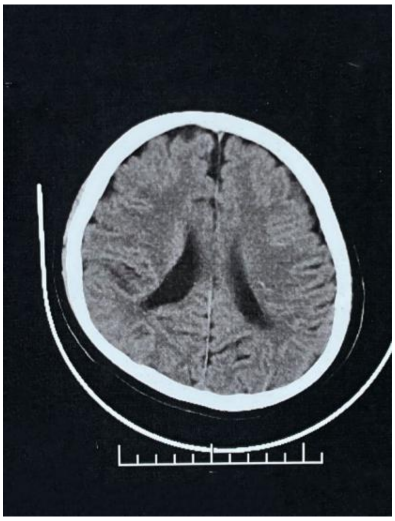
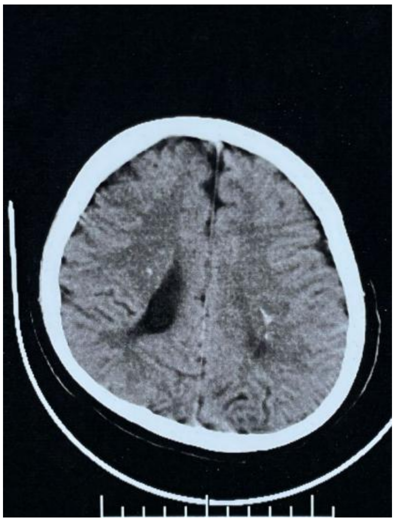
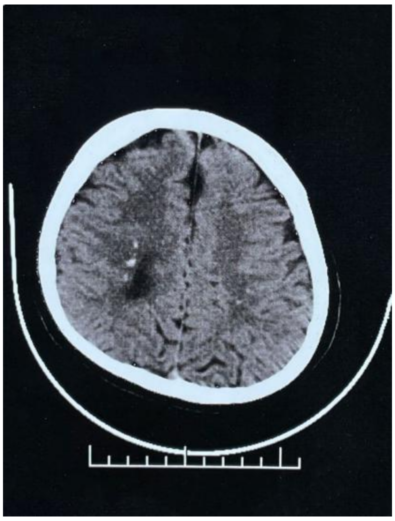
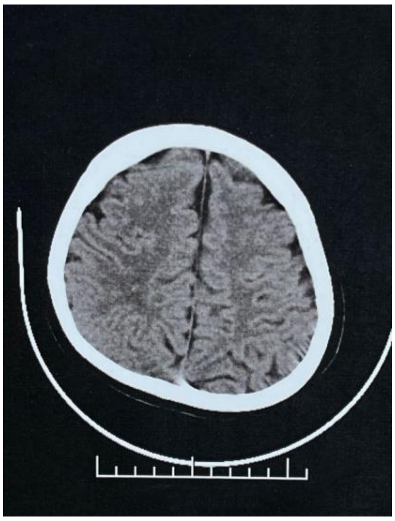
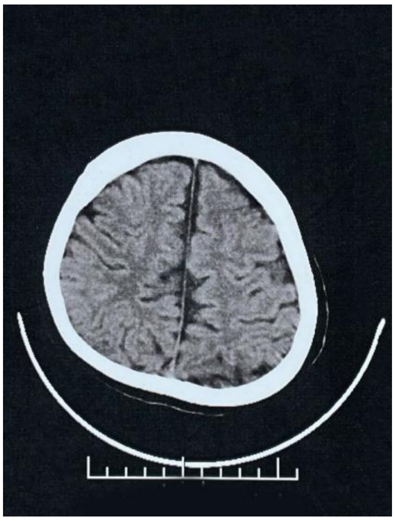
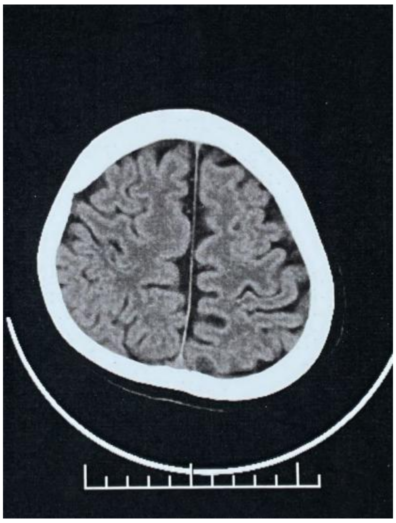
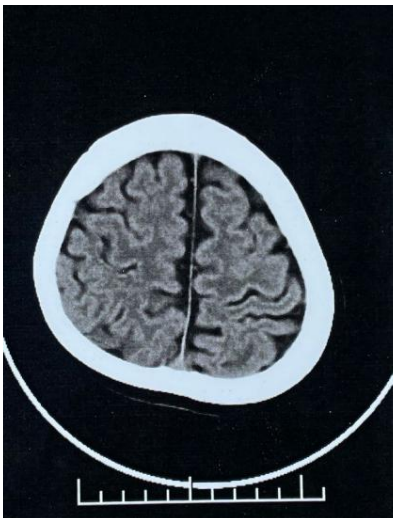
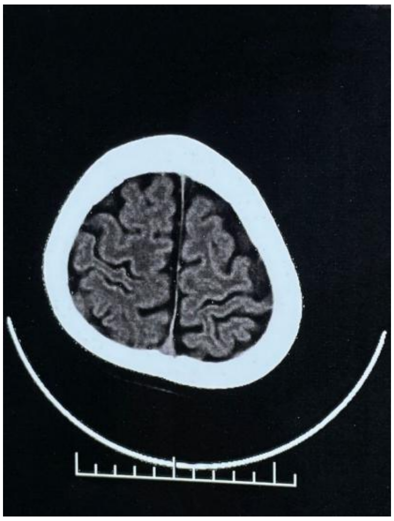
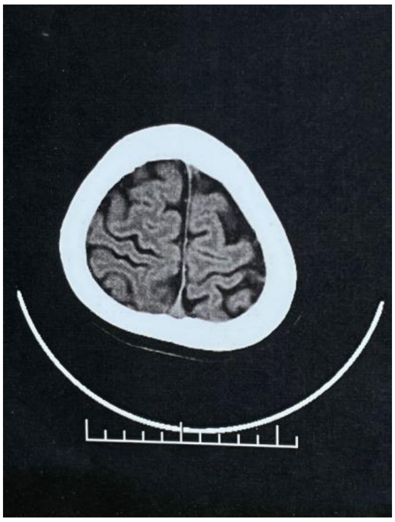

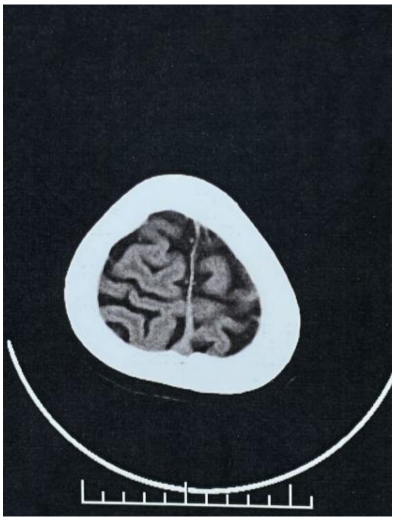

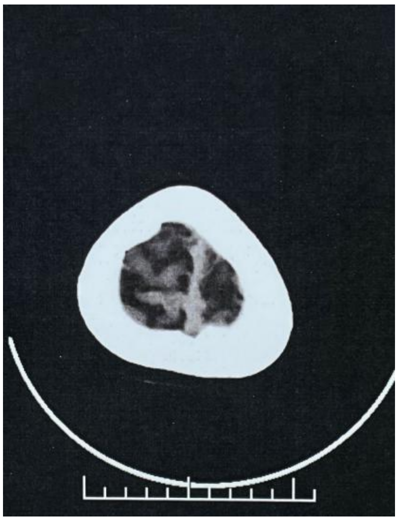

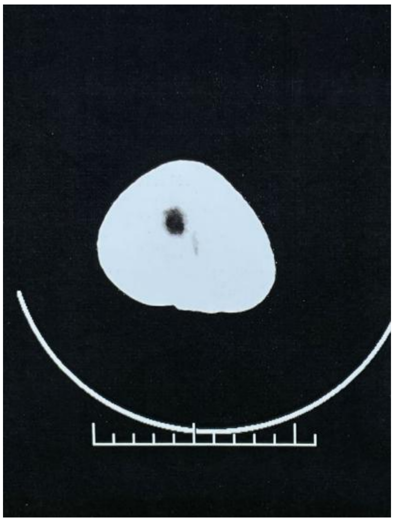

I. Brain CT scan images of individual III: 5

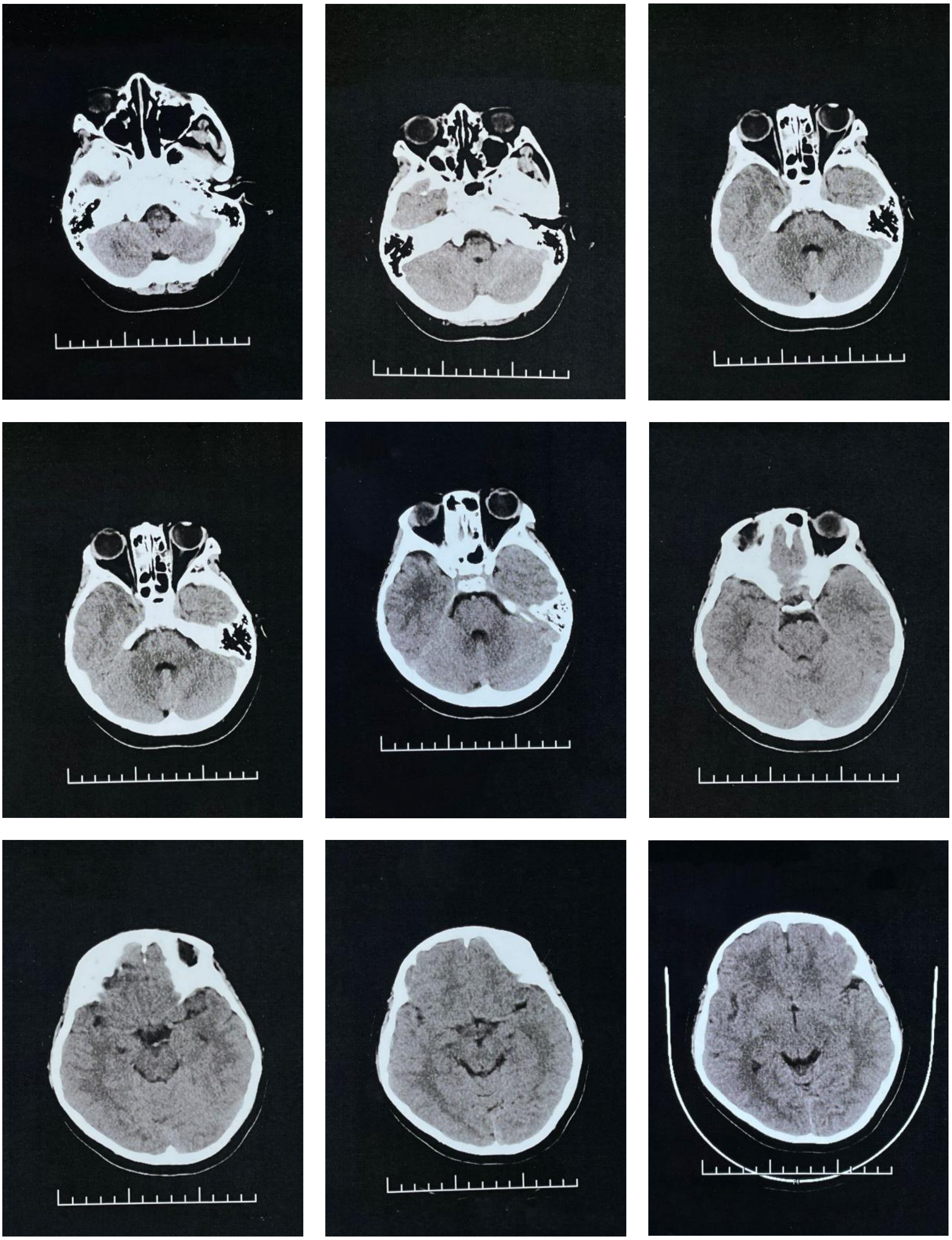

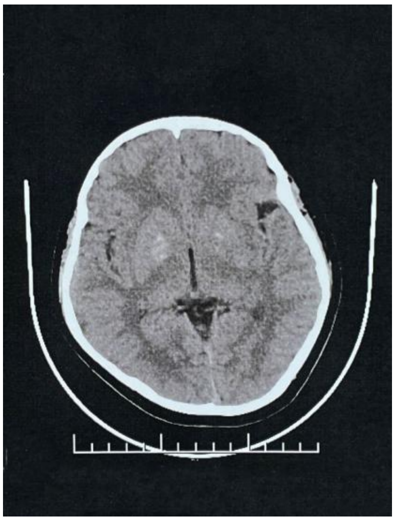
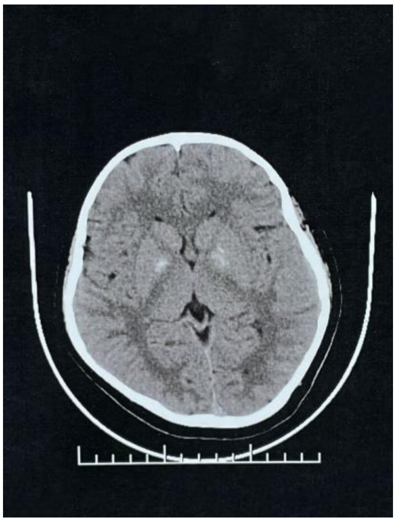
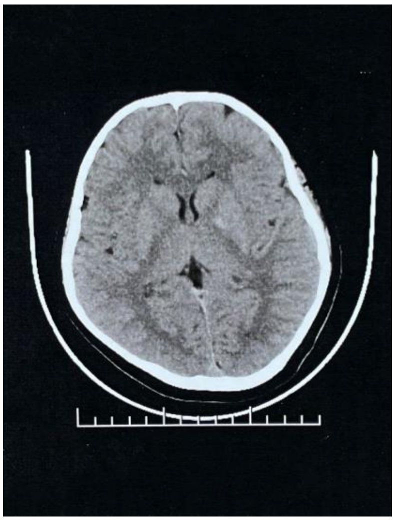
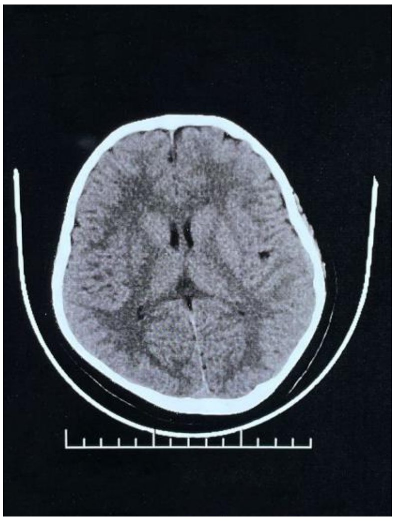
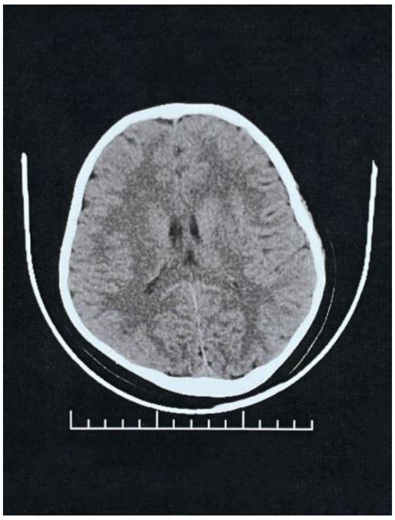
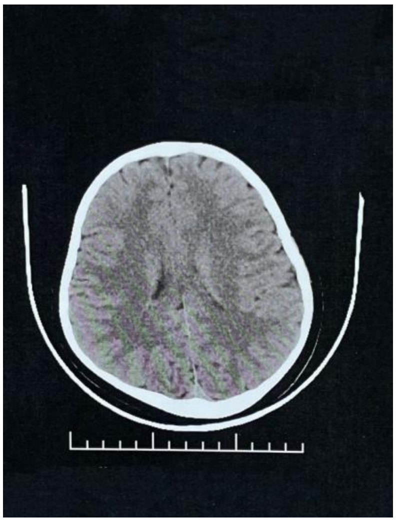
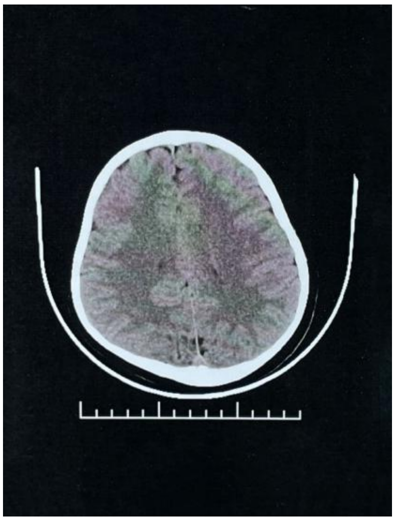
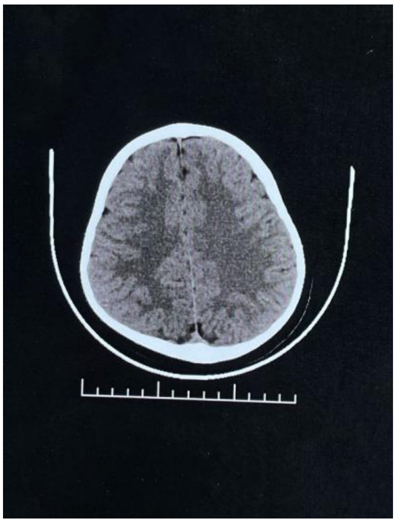
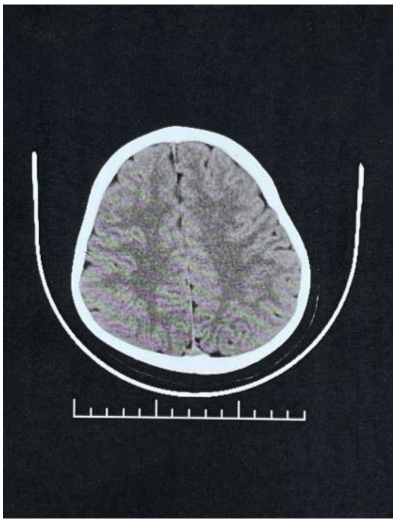

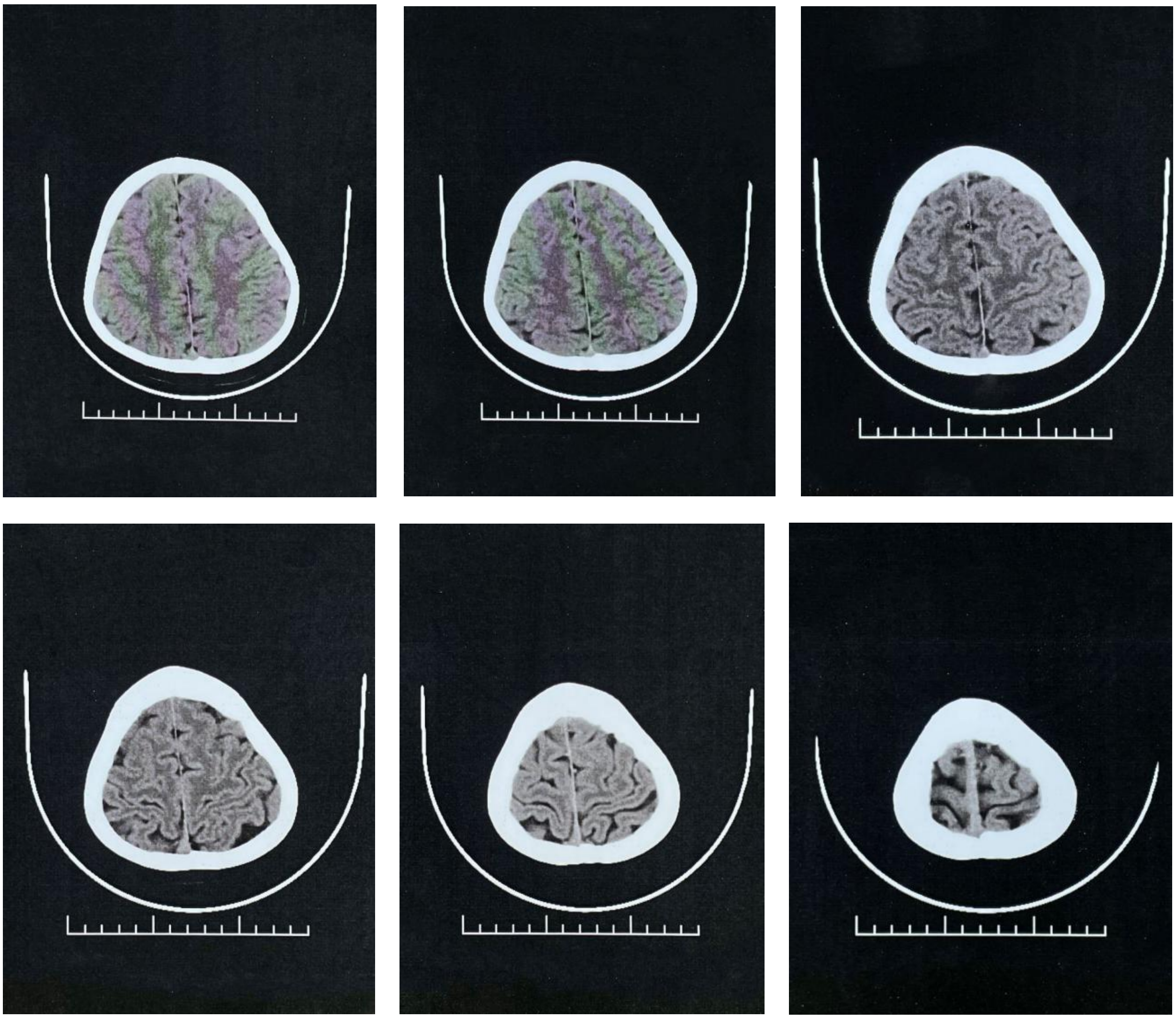

Supplement: Supplementary file 1 — Additional file 1. Brain CT scan images. A-I Brain CT scan images of individuals I: 1, I: 2, II: 1, II: 4, III: 1, III: 2, III: 3, III: 4, and III: 5. [file 12883_2022_2798_MOESM1_ESM.pdf]
