# Supplementary material for: SLC20A2-related primary familial brain calcification with purely acute psychiatric symptoms: a case report
Source: BMC Neurol. 2022 Jul 18;22:265. doi: 10.1186/s12883-022-02798-9 (PMC9290231; doi:10.1186/s12883-022-02798-9)

A. DNA sequencing results of individual I: 1

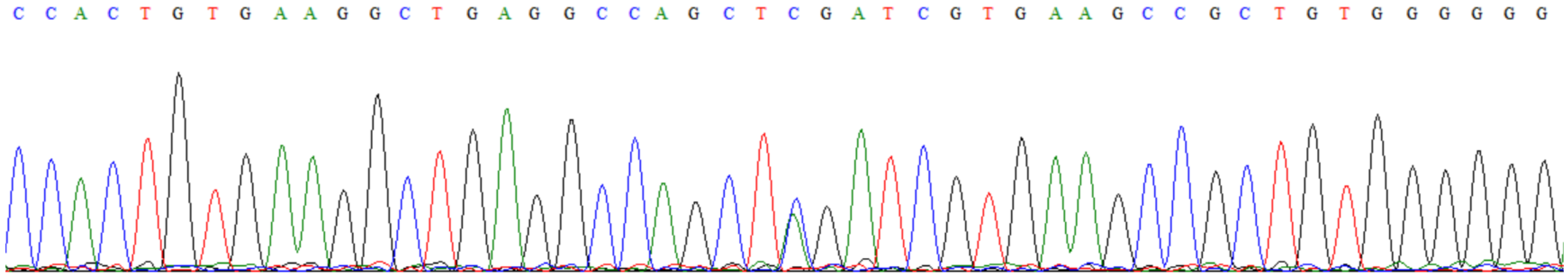

B. DNA sequencing results of individual I: 2

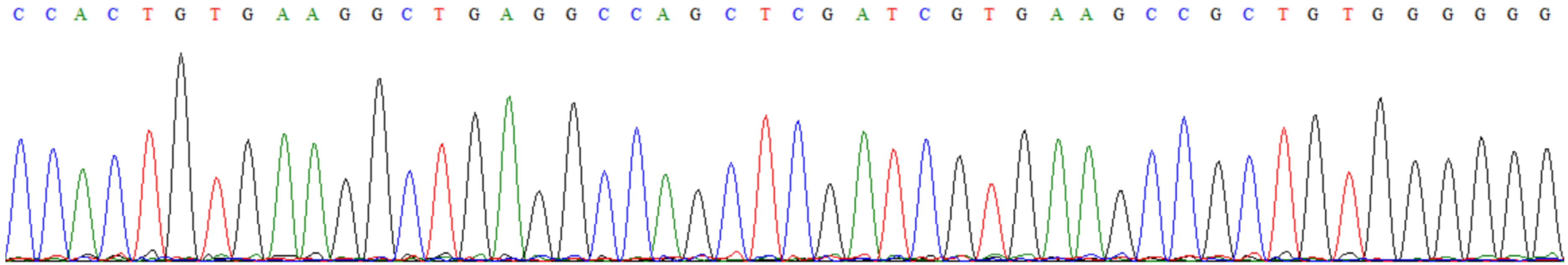

C. DNA sequencing results of individual II: 1

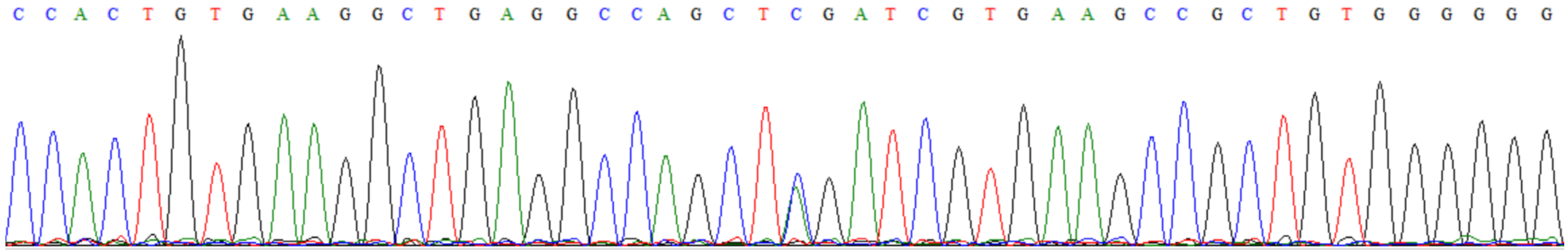

D. DNA sequencing results of individual II: 4

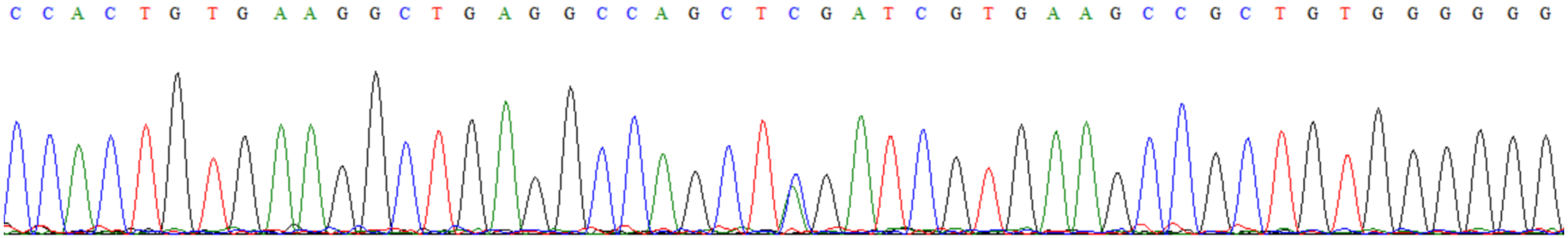

E. DNA sequencing results of individual III: 1

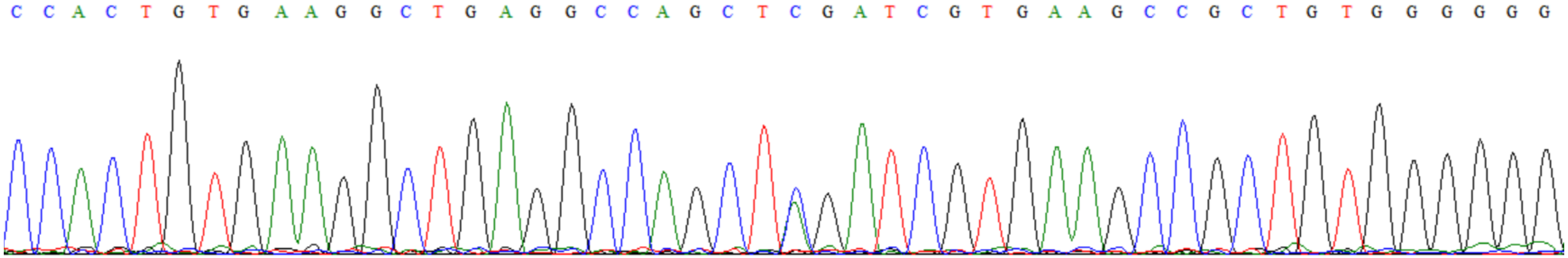

F. DNA sequencing results of individual III: 2

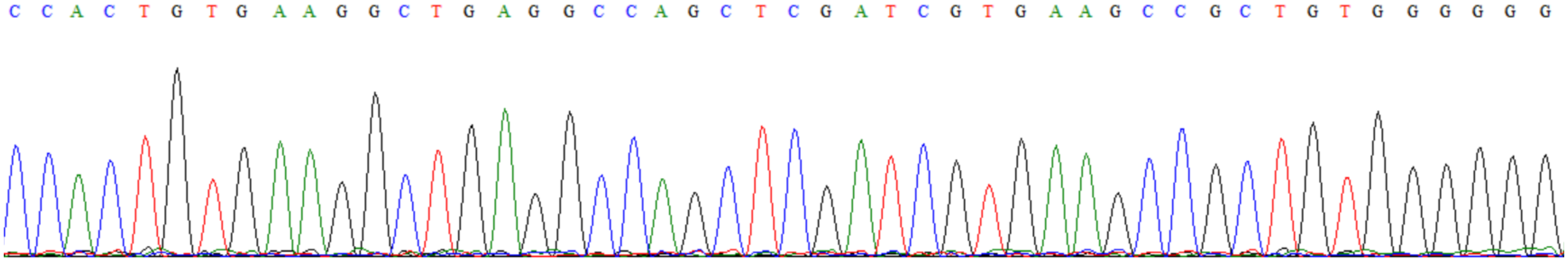

G. DNA sequencing results of individual III: 3

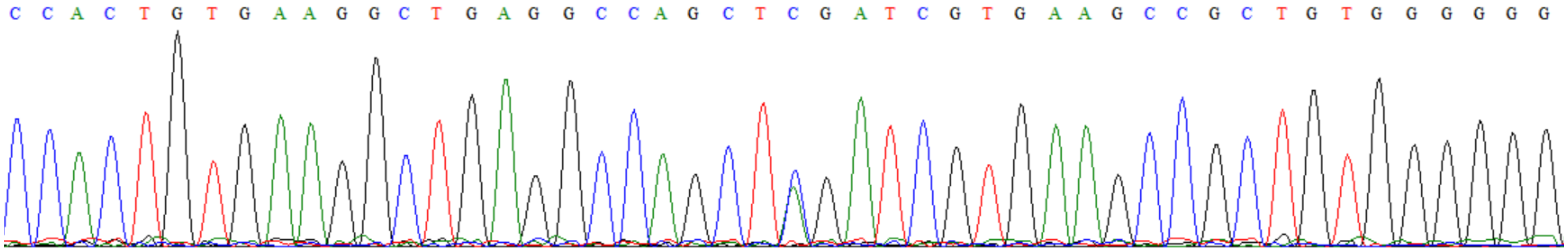

H. DNA sequencing results of individual III: 4

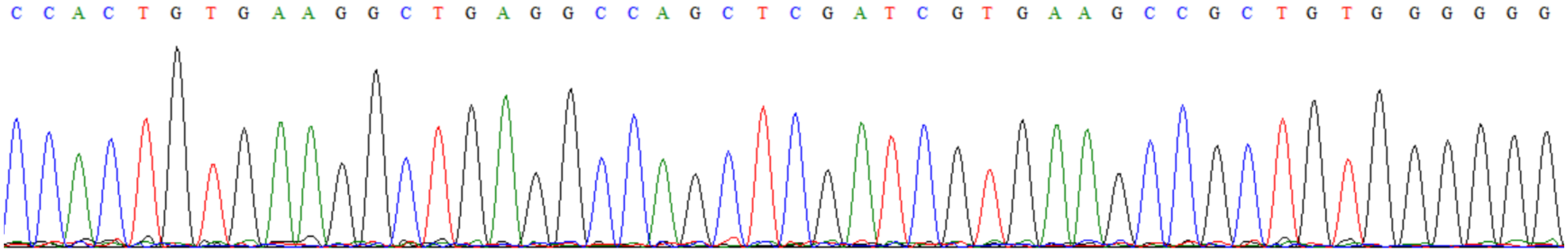

I. DNA sequencing results of individual III: 5

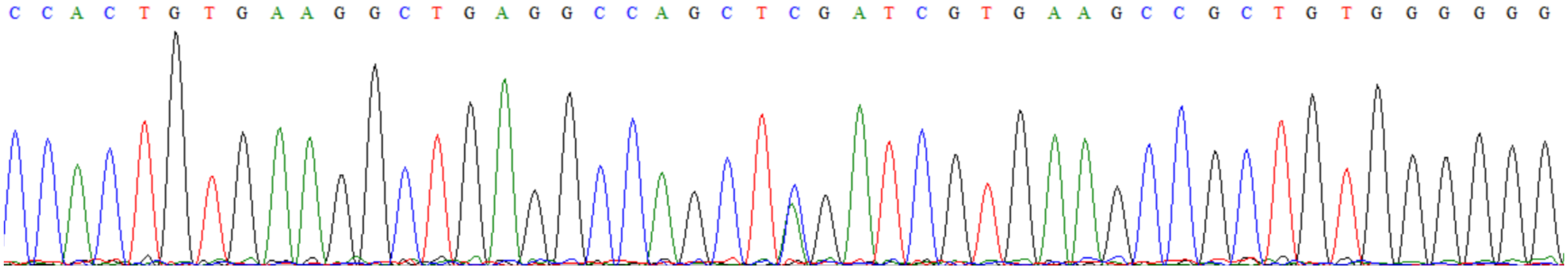

Supplement: Supplementary file 2 — Additional file 2. DNA sequencing results. A-I DNA sequencing results of individuals I: 1, I: 2, II: 1, II: 4, III: 1, III: 2, III: 3, III: 4, and III: 5. [file 12883_2022_2798_MOESM2_ESM.pdf]
